# 3D chemotaxis chip for investigating natural killer cell migration mechanisms

**DOI:** 10.3389/fimmu.2026.1762203

**Published:** 2026-08-13

**Authors:** Madison N. Temples, Suzanne Lightsey, Tiffany Conklin, Rylee S. Newport, Edward A. Phelps, Blanka Sharma

**Affiliations:** J. Crayton Pruitt Family Department of Biomedical Engineering, University of Florida, Gainesville, FL, United States

**Keywords:** biomaterials, cancer immunotherapy, cell migration, natural killer cells, three-dimensional, tumor microenvironment

## Abstract

**Introduction:**

Natural killer (NK) cells are a promising tool for cancer immunotherapy, as they can rapidly recognize and kill cancer cells without prior knowledge of tumor-specific antigens while leaving healthy cells unharmed. However, a major challenge in NK cell-based therapies is their inadequate infiltration and function within solid tumors. Advancements in NK cell therapies for solid malignancies require an understanding of the various factors that influence NK cell migration to and within the tumor microenvironment.

**Methods:**

In this study, we developed a chemotaxis chip with a tunable 3D hydrogel that enabled the spatiotemporal analysis of NK cell migration. Through live-cell imaging and cell-tracking analysis, we quantitatively assessed NK cell migration in engineered hydrogels in real time. This platform enabled precise control over matrix composition and physical properties, allowing systematic interrogation of microenvironmental features that regulate NK cell migration.

**Results:**

Our findings revealed that NK cells rely heavily on proteasedependent infiltration but can leverage alternative mechanisms for faster migration, and that the inclusion of hyaluronic acid, a tumorrelated extracellular matrix component, promotes NK cell migration in 3D.

**Discussion:**

This study established an adaptable hydrogel-based platform for studying immune cell migration in defined 3D environments, providing foundational tools for future mechanistic and translational investigations.

## 1 Introduction

Natural killer (NK) cells are potent effector cells of the innate immune system that eliminate cancer cells in an antigen-independent manner. Their mechanism of cancer cell recognition allows for the use of allogenic sources without the risk of graft-versus-host disease. As such, adoptive NK cell transfer has emerged as a promising therapy for cancer patients that bypasses several challenges associated with T cell-based therapies. NK cell-based treatments have shown both safety and efficacy in patients with advanced hematological malignancies (1, 2). However, progress in solid tumors has been markedly slower (3–5). Within solid tumors, NK cell infiltration is limited, and the cells that do infiltrate often remain in the tumor stroma (6–9), failing to establish the cancer cell-NK cell contact necessary to exert their cytotoxic function. Indeed, clinical studies suggest a predictive correlation between the abundance of NK cells in solid tumors and improved outcomes in patients with several types of cancer (10), including melanoma (11–13), breast cancer (14), non-small cell lung cancer (15), and others (16–18). Therefore, effective NK cell therapies that result in better infiltration and localization within the tumor are needed.

To reach a solid tumor, NK cells must first extravasate from the blood and traverse through the extracellular matrix (ECM) before exerting their cytotoxic functions (19). Recognition of chemokines secreted by tumors is critical for NK cell extravasation from the bloodstream and infiltration into the solid tumor (20). This is supported by the increased infiltration of engineered chemokine receptor-expressing NK cells into tumors (21–23), and the greater presence of NK cells in tumors that contain engineered chemokine-expressing cancer cells (24). However, NK cell infiltration is not dependent on chemokine secretion alone, as even with high levels of tumor-secreted chemokines, endogenous NK cell (25) and T cell (26) infiltration can be limited. Indeed, immune cells also rely on interactions with extracellular substrates to reach their destination; however, the extent or conditions under which the ECM acts as a substrate or a barrier to NK cell infiltration is unclear. For instance, in a syngeneic rat liver tumor model, NK cells could not infiltrate tumor nodules with containment structures composed of collagen IV and laminin, but could infiltrate nodules lacking such structures (27). Conversely, vitronectin in hepatic tumor ECM corresponded with greater leukocyte presence and retention (28), highlighting the role of integrin-mediated adhesion.

Hyaluronic acid (HA) is a major ECM component that is enriched in many solid tumors (29, 30) and can influence immune cell migration and retention (31, 32). Its prevalence and functional importance make HA a particularly relevant feature of the tumor ECM when considering factors that regulate NK cell motility. Other tumor microenvironmental characteristics, including hypoxia, acidic pH, and immunosuppressive cytokines, also impact immune cell migration, cytoskeletal dynamics, and functional engagement with target cells (33). Collectively, these ECM and microenvironmental cues likely shape NK cell infiltration and retention within solid tumors.

To understand the role of the tumor ECM on NK cell infiltration, NK cell migration mechanisms in tumor-relevant ECMs need further study. The two major modes of 3D cell migration are mesenchymal and amoeboid (34), with further classification in each category (35). Generally, the modes of migration are classified by their relative adhesion to the matrix and actin-myosin contractility. Cells using mesenchymal migration rely on actin polymerization at the leading edge to generate lamellipodia, and use integrins and associated focal adhesion proteins to interact with basement membranes (34, 35). Further, mesenchymal migration is largely dependent on matrix metalloproteinases (MMPs) to degrade the ECM (36). Conversely, amoeboid migration, which resembles the movement of protist amoeba, is conventionally MMP-independent, and driven by actin protrusion of hydrostatic membrane blebs (34, 35). Amoeboid migration requires high activity of Ras homolog gene family member A (RhoA) and rho-associated protein kinase (ROCK) for strong actin-myosin contractility to physically deform their cell body and squeeze through the surrounding pericellular matrix (34). While the effects of 3D ECM composition and architecture on immune cell migration has been well characterized in macrophages (37–39), dendritic cells (40–43), neutrophils (44–46), and T cells (47–50), much less is known about NK cell migration in 3D.

Conventional methods for evaluating human NK cell function have relied primarily on 2D cultures, which lack the three-dimensional structure and microenvironment of solid tumors. While these studies have provided insight into differences between resting and activated NK cells (51, 52) and the influence of surface topography on migration parameters (53), their findings must be interpreted with caution, as cell migration mechanisms in 3D differ substantially from those in 2D (40). To better mimic the tumor ECM, 3D systems using natural materials, such as collagen (51, 54–57), fibrin (58, 59), Matrigel (60), decellularized ECM (61, 62), have been used to study NK cell migration. These models enabled measurement of migration parameters such as speed, displacement, and persistence, advancing our understanding of NK cell behavior in tumor-like contexts. For example, microfluidic platforms revealed that tissue-resident NK cells outperform circulating NK cells in preventing metastatic seeding (62) and exhibit higher motility, traveling faster and infiltrating tumors more efficiently in 3D environments (59). However, the reliance on natural materials introduces inherent variability and compositional complexity, making it difficult to isolate the contributions of specific ECM features or to dissect distinct migration mechanisms (63, 64). These limitations highlight the need for reductionist, yet physiologically relevant, platforms in which biochemical and mechanical properties can be independently tuned.

Poly(ethylene glycol) (PEG) is one of the most widely studied synthetic polymers and can be modified with diverse functional groups. PEG hydrogels are often formed via photopolymerization, a non-toxic technique that supports cell encapsulation. Our previous work showed that NK92 cells, a clinically relevant NK cell line, migrate into PEG-based hydrogels using MMPs and integrins toward a chemotactic point source (83, 65). Unlike natural matrices, PEG hydrogels allow systematic tuning of ECM composition and stiffness, providing a systematic method to isolate the factors that regulate NK cell migration. This tunability overcomes the limitations of previous 3D models and supports real-time analysis of migration mechanisms.

In this work, we modified a chemotaxis chip to incorporate a PEG-based 3D hydrogel that allows precise control of biochemical properties (**Supplementary Figure S1**). The method enables real-time, quantitative analysis of NK cell motility and migration modes in response to defined matrix cues. By providing an engineered environment, the chemotaxis chip enables mechanistic studies that are difficult in natural matrices and serves as a versatile tool to uncover ECM-dependent migration strategies and enhance NK cell infiltration in solid tumors.

## 2 Materials and equipment

The chemotaxis chip was modified from the commercially available microfluidic device (Ibidi µ-Slide Chemotaxis) to support the migration of primary NK cells through an engineered 3D hydrogel. PEGdiacrylate (PEG-DA) and acrylate-PEG-succinimidyl valerate ester (acryl-PEG-SVA) were purchased from Laysan Bio Inc. The matrix metalloproteinase (MMP) degradable sequence, GGVPMS↓MRGGK, (MW 1076.31 Da) was purchased from Biomatrix. Cyclo(Arg-Gly-Asp-D-Phe-Lys) (RGD, MW 603.68 Da) and cyclo(Arg-Ala-Asp-D-Phe-Lys) (RAD, MW 617.71 Da) were purchased from Biosynth Inc. Dialysis tubing, Spectra/Por6–2000 MWCO, was purchased from Spectrum Laboratories. 2-Hydroxy-4’(2-hydroxyethoxy)-2-methylpropiophenone (Irgacure 2959) was purchased from Spectrum Laboratories. All inhibitors were purchased from Calbiochem. All other materials were obtained from Thermo Fisher Scientific, with specific concentrations and molecular weights detailed in the Methods section.

## 3 Methods

### 3.1 Primary cell isolation

Leukocyte reduction chambers from healthy donors were purchased from LifeSouth Gainesville after receiving informed consent from all participants. Peripheral blood mononuclear cells (PBMCs) were isolated via Ficoll centrifugation. NK cells were isolated from the PBMCs via negative selection magnetic separation (Untouched NK cells Dynabeads, Invitrogen). NK cells were cultured in RPMI 1640 (without L-glutamine), 10% fetal bovine serum, 1% penicillin-streptomycin, 1% L-glutamine, 1% non-essential amino acids, 1% sodium pyruvate, MycoZap, and 100 U/µL IL-2 (Peprotech, Inc.). NK cells were kept at a density of 1x10^6^ cells/mL in culture for 7 days at 37 °C and 5% CO_2_. Due to the limited yield and expansion capacity of primary NK cells, some donors were included in only a subset of experiments, with inclusion determined by the availability of sufficient viable cells. The study was approved by the University of Florida Institutional Review Board (IRB202002072). The demographics of donors are listed in **Supplementary Table 1**.

### 3.2 Flow cytometry

Viability, purity, NK cell subset, and chemokine receptor expression were assessed by flow cytometry after isolation and the subsequent days in culture. Cell viability was assessed by live/dead™fixable Aqua Dead Cell Stain Kit (Invitrogen). NK cell purity and phenotype were characterized by staining with conjugated with conjugated mouse anti-human antibodies against CD3 (Clone SK7, BD Biosciences), CD56 (Clone B159, BD Biosciences), and CD16 (Clone 3G8, BD Biosciences). NK cells chemokine receptors were characterized by staining with conjugated mouse anti-human antibodies against CXCR1 (Clone 5A12, BD Biosciences), CXCR2 (Clone 6C6, BD Biosciences), CXCR3 (Clone 1C6/CXCR3, BD Biosciences), and CXCR4 (Clone 12G5, BD Biosciences). NK cells are defined as CD3- and CD56+ (representative gating strategy shown in **Supplementary Figure S2**). A minimum number of 100,000 cells were analyzed using a BD FACS Celesta Flow Cytometer and Flowjo Software v10 (FLOWJO, LLC Data analysis software).

### 3.3 Fabrication of chemotaxis chip

The device consists of three individual chambers, each with two symmetrical reservoirs connected by a narrow observation channel. Our experimental design included one reservoir with NK cells, the other containing medium (either with or without CXCL11), and the observation channel containing the hydrogel, unless specified otherwise. Briefly, 5.12 µL of the hydrogel precursor solution was injected to the top port of the observation chamber with the ports plugged on the outer reservoirs as detailed by Ibidi. 6 µL of air was aspirated from the bottom port of the observation chamber to pull the precursor solution through. This process was repeated for all three chambers. The slide was then photopolymerized by the OmniCure S1000 light (Excelitas Technologies, Corp.) for 3 minutes using 4.0 mW/cm^2^ long wave ultraviolet A (UVA) light. For donors 5-7, the slide was photopolymerized by OmniCure S2000 light (Excelitas Technologies Corp.) for 2.5 minutes using 4.24 mW/cm^2^ long wave UVA light. The hydrogels were washed by removing the plugs from the reservoirs and filling the ports with 65 µL of PBS. Then the PBS was removed from one reservoir and 65 µL of NK cell culture medium was added via the top port. Then, the PBS was removed from the opposite reservoir and 75 µL of NK cells suspended in medium (at 4x10^6^ cells/mL) was added via the top port. Lastly, 10 µL of 1 ng/µL CXCL11 in medium or medium alone, for the control, was added to the top port of the reservoir containing media only. This CXCL11 concentration was selected based on prior 3D NK cell migration studies and falls within the range reported for NK cell-active chemokines from tumor cells, providing a physiologically relevant gradient to trigger measurable migration *in vitro* (83, 66). For studies quantifying the concentration gradient, FITC-dextran was used instead of CXCL11.

To assess CXCL11-hydrogel interactions, CXCL11 was encapsulated in 10 µL hydrogels containing MMP-degradable sites, RAD or RGD motifs (5 mM), or MeHA (500 µg/mL). Hydrogels were incubated in 200 µL of PBS at 37 °C and 5% CO_2_ for 24 hours, and the supernatant was collected. CXCL11 concentration in the supernatant was quantified via ELISA (R&D Systems, Cat. DCX110) to compare release across different hydrogel compositions (**Supplementary Figure S3**).

**Figure 1:**
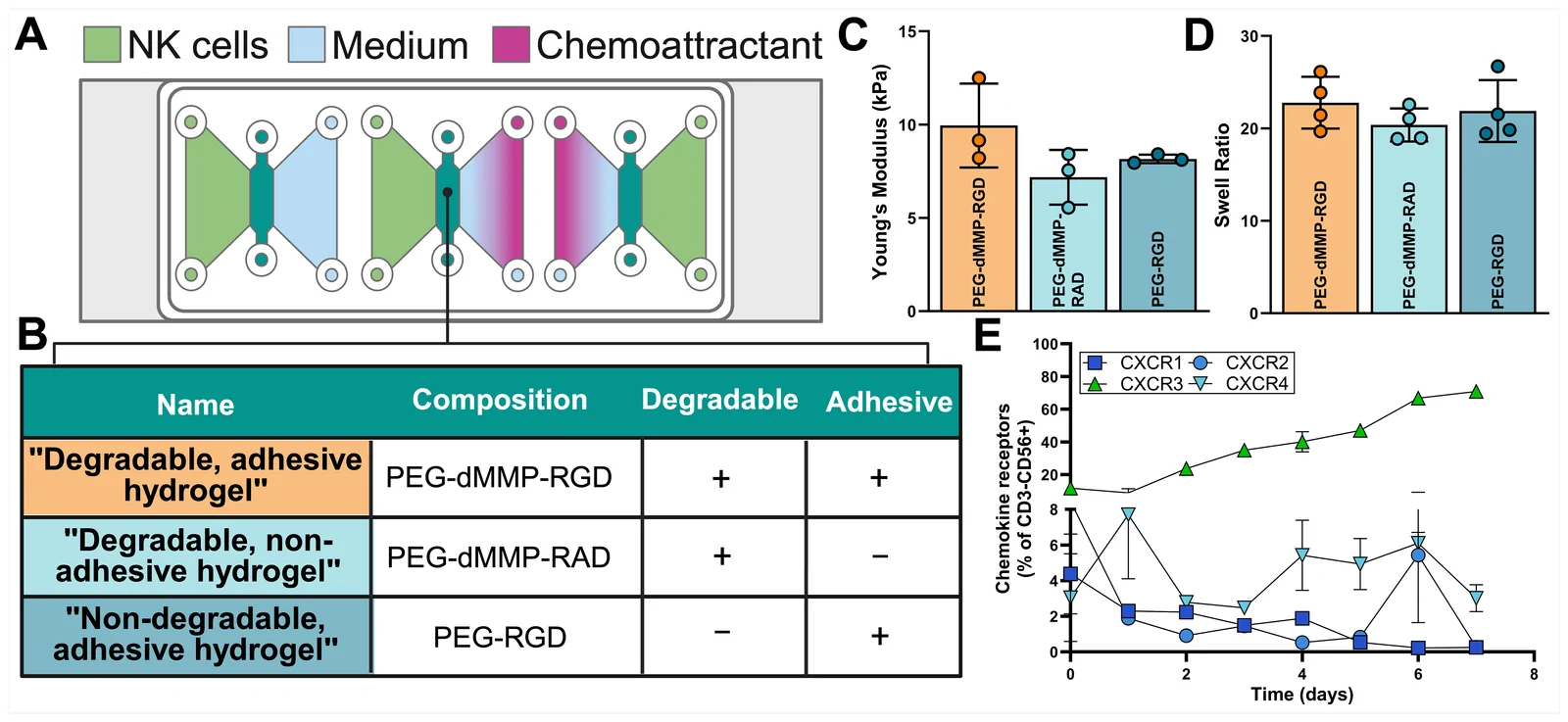
Establishment of the chemotaxis migration chip. **(A)** Schematic detailing an Ibidi chemotaxis µ-Slide chip set-up, with a hydrogel in the middle observation chamber. **(B)** Engineered PEG-based hydrogels and their properties which were crosslinked in the middle chamber of the chemotaxis chip. **(C)** Characterization of the stiffness of engineered hydrogels. **(D)** Quantification of the swell ratio of engineered hydrogels. **(E)** Characterization of primary NK cell chemokine receptor expression of CD3^-^CD56^+^ NK cells. Results are one donor representative of three donors.

Primary NK cells were seeded in their respective reservoirs one or two days after isolation. Each chemotaxis chip would investigate one experimental parameter with the following set up: chamber 1 had NK cells in the left reservoir and medium only (control) in the right reservoir, chamber 2 had NK cells in the left reservoir and CXCL11 in the right reservoir, and chamber 3 had NK cells in the right reservoir and CXCL11 in the left reservoir (**Figure 1A**). Having the chemokine in different reservoirs provides further validation by accounting for any external tilt the chip may experience during culture or imaging.

### 3.4 Cell culture in chemotaxis chip

The chemotaxis chip was cultured at 37 °C and 5% CO_2_. Primary NK cells were expanded and maintained in IL-2 prior to and during the migration assays to preserve viability, cytotoxic function, and migratory capacity. IL-2 activation can enhance NK cell metabolic activity and chemokine receptor expression, supporting effective migration (67). Clinically, IL-2 is often co-administered with NK cell therapies to support *in vivo* survival and function, making this activation state physiologically relevant. Importantly, all experimental conditions used NK cells under the same IL-2 activation regimen, ensuring that relative comparisons between hydrogel conditions were not confounded by differences in activation state. While in culture, the media and the chemoattractant gradient were refreshed daily. The medium was removed from the individual CXCL11 or control reservoirs and 65 µL of NK cell culture medium was added to each of the CXCL11 or control reservoirs via the top port. Then, 75 µL of NK cell culture medium was added to the NK cell-containing reservoirs via the top port and approximately 60 µL of spent NK cell culture medium was removed from the bottom port. Lastly, 10 µL of CXCL11 in medium (1 ng/µL) or medium alone for the control group was added to the top port of the medium containing reservoirs. Chemotaxis chips were imaged using a Leica TCS SP8 confocal microscope (Leica Microsystems) on day 5 of culture, with media and chemokine refreshed at least one hour prior to imaging to allow cells to recover from any transient mechanical perturbations. To control for effects of media handling, wells without CXCL11 were included as controls, enabling the distinction between true chemotactic responses and potential perturbations from the media change.

### 3.5 Peptide functionalization and polymer synthesis

The MMP-sensitive peptide sequence VPMS↓MRGG was included in the hydrogel to support cell migration. The peptide was modified to be capped on either end of a PEG arylate group upon conjunction as previously described (83, 68–71). Briefly, the terminal and lysine amine groups of the MMP degradable sequence were reacted with the SVA of an acryl-PEG-SVA in 50 mM sodium bicarbonate (pH = 8.0) at a 1:2 molar ratio for 4 hours. The reaction product was dialyzed for 24 hours against water to remove unreacted reagents, lyophilized for 48 hours and stored at -20 °C until use.

Cell adhesion was supported by immobilizing cell adhesion ligands to the crosslinked network. RGD functionalized PEG, acryl-PEG-RGD (PEG-RGD, MW 4005 Da), which mimics the adhesion site on vitronectin (72–74), was synthesized as previously described (69–71). Briefly, the terminal amine of cyclo(ArgGly-Asp-D-Phe-Lys) (cRGD) reacted with acryl-PEG-SVA in 50 mM sodium bicarbonate (pH = 8.0) at a 1.05:1 molar ratio for 4 hours. The reaction product was dialyzed for 24 hours against water to removed unreacted reagents, lyophilized for 48 hours, and stored at -20 °C until use. Acryl-PEG-RAD (PEG-RAD, MW 4013 Da), a control with no integrin binding site was synthesized in the same manner, by reacting the terminal amine of cyclo(Arg-Ala-Asp-D-Phe-Lys) with the SVA of an acryl-PEG-SVA.

Methacrylated hyaluronic acid (MeHA) was synthesized as previously described (75). Hyaluronic acid (HA) was dissolved in a 50:50 ratio of PBS and acetone solution at 10 mg/mL overnight. Triethylamine was added and stirred for 30 minutes, followed by the addition of glycidyl methacrylate and stirred overnight. The stoichiometric ratio of glycidyl methacrylate added to each HA monomer was 20X. MeHA solution was precipitated by pouring the dissolved solution into a large beaker of acetone and stirring with a glass rod to collect the precipitate. Once the solution precipitated, MeHA was rinsed with fresh acetone and dissolved in PBS overnight, followed by another precipitation in acetone. MeHA solution was then dialyzed for 72 hours against PBS, followed by another dialysis for 36 hours against water. MeHA solution was sterile filtered, flash frozen, lyophilized for 5 days, and stored at -20 °C until use. This process was done for high molecular weight HA (750–1000 kDa, bacteria-derived, Lifecore Biomedical) and low molecular weight HA (10–30 kDa, bacteria-derived, Sigma Aldrich) (30).

### 3.6 Peptide functionalization analysis

The PerkinElmer Frontier spectrometer was utilized to obtain the attenuated total reflectance Fourier transform infrared spectra (ATR-FTIR) of lyophilized PEG-dMMP-PEG, PEG-RGD, and PEG-RAD. The spectrometer was equipped with desiccate Ga-coated KBr optics, a temperature-stabilized deuterated triglycine sulfate detector, and one reflection diamond/ZnSe universal ATR sampling accessory (76). ATR-FTIR selected characteristic bands for PEG-SVA: C-H stretching 2880 cm^-1^ and C-O stretching 1102 cm^-1^. ATR-FTIR selected characteristic bands for cRGD peptide: aromatic ring 1644 cm^-1^. ATR-FTIR selected characteristic bands for dMMP peptide: C=O stretching 1645 cm^-1^ and C-S stretching 660 cm^-1^. Confirmation of PEG functionalized for PEG-dMMP-PEG and PEG-RGD are shown in **Supplementary Figures 4**, **5**, respectively.

The degree of methacrylation of HMW and LMW MeHA was calculated via proton nuclear magnetic resonance (HNMR) spectroscopy. HNMR spectra were obtained using a 600 MHz Bruker Avance III spectrophotometer. Deuterium oxide (ACROS Organics) was used as a solvent and analysis was done at room temperature. The relative peak integration was performed between methacrylate protons (peak at 1.8 ppm) and methyl protons already present on the HA backbone (peak at 1.9 ppm), as previously described (75). This is referenced as the “degree of methacrylation” to mean the percentage of HA monomers containing methacrylated groups. For HMW and LMW MeHA, the degree of methacrylation was 21.27% and 19.97% (**Supplementary Figure S6**).

### 3.7 Characterization of hydrogel properties

The precursor solutions for the different hydrogel formulations, made in PBS, are listed in **Supplementary Table 2**. The photoinitiator, Irgacure 2959, was added to each precursor solution (0.05% w/v) before polymerization. Briefly, hydrogels were formed in cylindrical silicon molds (Grace Biolabs) 8 mm diameter by 1.7 mm depth. The precursor solution was added to the silicon mold and photopolymerized by the OmniCure S2000 light for 3 minutes at 4.24 mW/cm^2^ long wave UVA light. After polymerization, the hydrogels were incubated in PBS at 37 °C and 5% CO_2_ for 24 hours. The viscoelastic properties were examined using an Anton Paar MCR 302 rheometer with parallel plate geometry according to established methods (77, 78). An 8 mm sandblasted top load cell was used to decrease slipping during testing. For an approximation of physiological conditions, the Peltier plate was preheated to 37 °C, and the samples were enclosed in a humidity chamber. For each hydrogel composition, amplitude sweeps from 0.01% to 100% strain were first conducted to determine the linear viscoelastic region (LVE). The storage (G’) and loss (G”) moduli were then determined from frequency sweeps from 0.1 to 100 Hz at the strain value in the middle of the LVE, chosen as 0.1% strain for all hydrogel compositions. For all hydrogel compositions, shear moduli (G*) were determined from Equation 1 at 1 Hz and used to calculate the Young’s modulus (E) using Equation 2 with a Poisson’s ratio (*υ*) of 0.5 (108, 79–82). For each condition, three samples were used for the amplitude sweeps and four samples were used for the frequency sweeps, wherein each hydrogel was used only for one test.

(1) $$G^{*}=\sqrt{G{'}^{2}-G{”}^{2}}$$

(2) $$E=2G^{*}(1+\upsilon )$$

The swell ratios of the hydrogels were determined as previously described (83). Precursor solutions (10 mL) were photopolymerized as previously described. After polymerization, the hydrogels were incubated in PBS for 24 hours at 37 °C and 5% CO_2_. The hydrogels were carefully blotted with a Kim wipe to remove excess surface liquid, and the swollen wet weight (m*_s_*) was measured. Hydrogels were lyophilized for 24 hours and the dry weight of that hydrogel (m*_d_*) was measured. The swell ratio at 24 hours was calculated by Equation 3.

(3) $$\text{Swelling}\;\text{Ratio}=\frac{m_{s}-m_{d}}{m_{d}}$$

Further, for each composition, the weight fractions of the gel after crosslinking (q*_F_*, 0 hours) and when swollen (q*_W_*, 24 hours) were determined by Equations 4, 5, respectively. Here, m*_c_*is the wet weight of the hydrogel immediately after crosslinking and m*_d_*is the dry weight of that hydrogel, and m*_s_*is the swollen gel wet weight and m*_d_*is the dry weight of that hydrogel.

(4) $${\text{q}}_{F}=\frac{m_{c}}{m_{d}}$$

(5) $${\text{q}}_{W}=\frac{m_{s}}{m_{d}}$$

Additionally, it was necessary to calculate the volume fraction of the polymer of the swollen gel (*υ*_2,s_) and the volume fraction of the polymer after crosslinking (*υ*_2,r_) by Equations 6, 7. Here, the density of PEG (*ρ_p_* = 1.12 g/cm^3^) and the density of the solvent, PBS (*ρ*_sol_=1.00 g/cm^3^) are needed for the calculations.

(6) $$\phi =\upsilon_{2,\text{s}}={\left[1+\frac{(q_{W}-1)\rho_{p}}{\rho_{\text{sol}}}\right]}^{-1}$$

(7) $$\upsilon_{2,\text{r}}={\left[1+\frac{(q_{F}-1)\rho_{p}}{\rho_{\text{sol}}}\right]}^{-1}$$

The molecular weight between crosslinks, M*_C_*, can be calculated by the Peppas-Merrill equation, shown in Equation 8 (84–86). The Peppas-Merrill equation is a modification of the Flory-Rehner equation and applies to swollen networks of crosslinked polymers prepared under conditions that the crosslinks were introduced in solution (84–86). Here, the number-average molecular weight of the polymer (M*_n_*) for PEG-dMMP-PEG was 7900 Da, the specific volume of the polymer (*υ* for PEG was 0.893 cm^3^/g (87, 88), the molar volume of solvent (V_1_, for water) was 18 g/mol (86), and the polymer solvent thermodynamic interaction parameter (*χ*) for PEG and water was 0.43 (87–89).

(8) $$\frac{1}{M_{C}}=\frac{2}{M_{n}}-\frac{\bar{\upsilon }/V_{1}[ln\;(1-\upsilon_{2,s})+\upsilon_{2,\text{s}}+\chi {\left(\upsilon_{2,\text{s}}\right)}^{2}}{\left(\upsilon_{2,\text{r}})[\frac{\upsilon_{2,\text{s}}^{1/3}}{\upsilon_{2,\text{r}}}-\frac{\upsilon_{2,\text{s}}}{2\upsilon_{2,\text{r}}}\right]}$$

The mesh sizes of the hydrogels were then determined by Equations 9, 10 (85, 90). The average end-to-end distance of the polymer chain in the unperturbed state [ ${\left({\bar{r}}_{0}^{2}\right)}^{1/2})$] was determined by Equations 4-9, where the molecular weight of the repeating unit (M*_r_*) for PEG was 44, the average bond length (l) for PEG was 0.154 nm (90) and the Flory characteristic ratio of the polymer (C*_n_*) for PEG was 4.0 (87, 88).

(9) $${\left({\bar{r}}_{0}^{2}\right)}^{1/2}=lC_{n}^{1/2}{\left(2\frac{M_{C}}{M_{r}}\right)}^{1/2}$$

(10) $$\xi =\upsilon_{2,\text{s}}^{-1/3}{\left({\bar{r}}_{0}^{2}\right)}^{1/2}$$

### 3.8 NK cell inhibition

For studies involving inhibitors, NK cells were pre-treated for 2 hours with the inhibitor and cultured in inhibitor-containing medium for the duration of the experiment. The inhibitors did not affect NK cell viability (**Supplementary Figure S7**). In all inhibitor studies, the degradable, adhesive hydrogel (PEG-dMMP-RGD) was used. To inhibit MMPs, NK cells were treated with 5 µM of the broad-range MMP inhibitor GM6001. To inhibit integrin binding, NK cells were treated with 50 µM of a soluble RGD peptide. To inhibit ROCK, NK cells were treated with 20 µM of Y27632.

### 3.9 Confocal microscopy and image analysis

Chemotaxis chips with primary NK cells were imaged using a Leica TCS SP8 confocal microscope with HC PL APO CS2 10x/0.4 Dry air objective. The microscope was pre-warmed to 37 °C and pre-set to 5.5% CO_2_, the humidity chamber had a dish filled with synergy water and two Kim wipes rolled up and soaked with synergy water to prevent evaporation. Additionally, chemotaxis chips were taped down on all sides (avoiding the observation areas) so that the chip would not shift during imaging. NK cell migration in the chip was imaged with spatial temporal time-course imaging, which was acquired at 512 X 512-pixel resolution. Chemotaxis chips were imaged for 1.5–2 hours, with images being acquired every 2 minutes. This imaging duration was selected to capture key migration parameters, including velocity, displacement, and directionality, while balancing practical constraints of time-lapse imaging. Although slower-moving cells may not be fully captured in this window, prior studies have shown that the majority of migration behavior can be reliably quantified within 90 minutes (91). Images were acquired with a pinhole of 11.31, a zoom of 4.00, and a z-stack size of 10-25 µm. FITC-dextran diffusion chips were imaged with spatial temporal time-course imaging, which was acquired at 512 X 512-pixel resolution. The chips were imaged for 24 hours, with images being acquired every 30 minutes. Images were collected with a pinhole of 11.31, a zoom of 1.25, and a z-stack size of 100 µm. For all studies, the z-stack images were projected onto a 2D image using volume intensity projections (**Video S1** and **S2**).

Image analysis was performed using Fiji (National Institute of Health) (92). NK cells in the hydrogel at time 0, the first image of the series, were counted using the Cell Counter plugin. Then the cell tracks were manually tracked with the Manual Tracking plugin in Fiji (National Institute of Health) for the first 90 minutes of migration. The CSV file with x and y locations from the Manual Tracking plugin were then imported into a Wolfram Mathematica code developed to take those tracks and determine migration parameters. First, the cell tracks were adjusted so the (x, y) position was (0, 0) at 0 hour for each cell. From these modified cell tracks the accumulated distance, Euclidean distance, and velocity were evaluated for each cell. The accumulated distance is an average of all the cells movements measured in between all analyzed images (slices), m, calculated by Equation 11, di,j is the displacement of the cell number I from image j-1 to image j. The Euclidean distance is an average of all the cells length of the individual cell start and end and calculated by Equation 12. The velocity (V) is calculated by Equation 13. Only tracks with an accumulated distance of 20 µm (2x the cell body) or more were included in the results.

(11) $$d_{i,accum}=\frac{1}{m}\sum_{j=2}^{m}d_{i,j}$$

(12) $$d_{i,euclid}=\sqrt{{\left(X_{i,end}-Xi,start\right)}^{2}+{\left(Y_{i,end}-Y_{i,start}\right)}^{2}}$$

(13) $$V=\frac{d_{i,accum}}{time}$$

### 3.10 Statistical analysis

GraphPad Prism V9 (GraphPad Software, San Diego, CA, USA) was used for data visualization. To assess the role of CXCL11 in the hydrogel, a paired Student’s t-test was performed. To account for the hierarchical structure of the primary NK cell migration data (multiple cells per donor), a linear mixed-effects model was fitted in R using the lmer function from the lme4 package. Cell migration parameters were specified as the dependent variables, with experimental group included as a fixed effect and donor identity as a random intercept to account for the non-independence of cells derived from the same donor (Equation 14), where *y_ij_* is the migration parameter for cell *i* from donor *j*, group*_ij_*denotes the experimental group, *β*_0_ is the intercept, *β*_1_ is the fixed effect of group, *b_j_*is the random intercept for donor *j*, and *∈_ij_*is the residual error term. The random effects are assumed to follow $b_{j}∼N(0,\sigma_{b}^{2})$ and $\in_{ij}∼N(0,\sigma^{2})$.

(14) $$y_{ij}=\beta_{0}+\beta_{1}\;{\text{group}}_{ij}+b_{j}+\in_{ij}$$

Effect sizes are reported as the estimated difference between groups (∆) obtained from the model, together with the corresponding p values. Model assumptions were assessed by visual inspection of residual distributions using quantile–quantile (QQ) plots of model residuals (**Supplementary Figure S8**). Statistical significance was defined as p *<* 0.05.

Not all experiments included all nine NK cell donors. For velocity, accumulated distance, and Euclidean distance plots, each small point represents an individual cell, while larger circles indicate the corresponding donor mean.

## 4 Results

### 4.1 Development of a chemotaxis chip for 3D NK cell migration

We developed a 3D migration assay by modifying the commercially available µ-Slide Chemotaxis, a flow-free microfluidic tool amenable to time-lapse microscopy, to include a 3D PEG hydrogel in the observation area (**Figure 1A**). Briefly, the µ-Slide Chemotaxis features three separate chambers, with each chamber containing two symmetrical reservoirs bridged by a narrow observation channel. The observation chamber was filled with the desired PEG precursor solution (**Figure 1B**; **Supplementary Figure S1**), polymerized using UVA light, and the reservoirs were filled with medium containing either primary NK cells or the chemoattractant. This setup allows for time-lapse imaging of NK cells migrating through the hydrogel.

We engineered three biochemically distinct PEG-based hydrogels with varying degrees of adhesivity and degradability to evaluate specific mechanisms of NK cell migration (**Figure 1B**). These formulations had similar mechanical properties, as assessed by rheometry and hydrogel swelling properties (**Figures 1C, D**). Across all hydrogel formulations, the measured Young’s modulus was 8.42 ± 1.8 kPa, with no statistically significant differences observed between groups.

Primary NK cells were isolated via negative selection from peripheral blood mononuclear cells of healthy human donors. Donor demographics are listed in **Supplementary Table 1**. After isolation, the NK cells remained viable, were free of contaminating T cells, and had a high purity of CD3^-^CD56^+^ cells (**Supplementary Figure S9**). To determine the appropriate chemokine to characterize NK cell migration, we explored the expression of chemokine receptors on the isolated NK cells. Of the chemokine receptors studied, CXCR3 was the most prevalent on primary NK cells both after isolation and after 7 days of culture (**Figure 1E**). Based on the expression of the chemokine receptors on the isolated NK cells, CXCL11 was selected as the chemoattractant for subsequent migration assays, as this is the most potent ligand for CXCR3 (93).

### 4.2 Characterization of the concentration gradient in the chemotaxis chip

**Figure 2:**
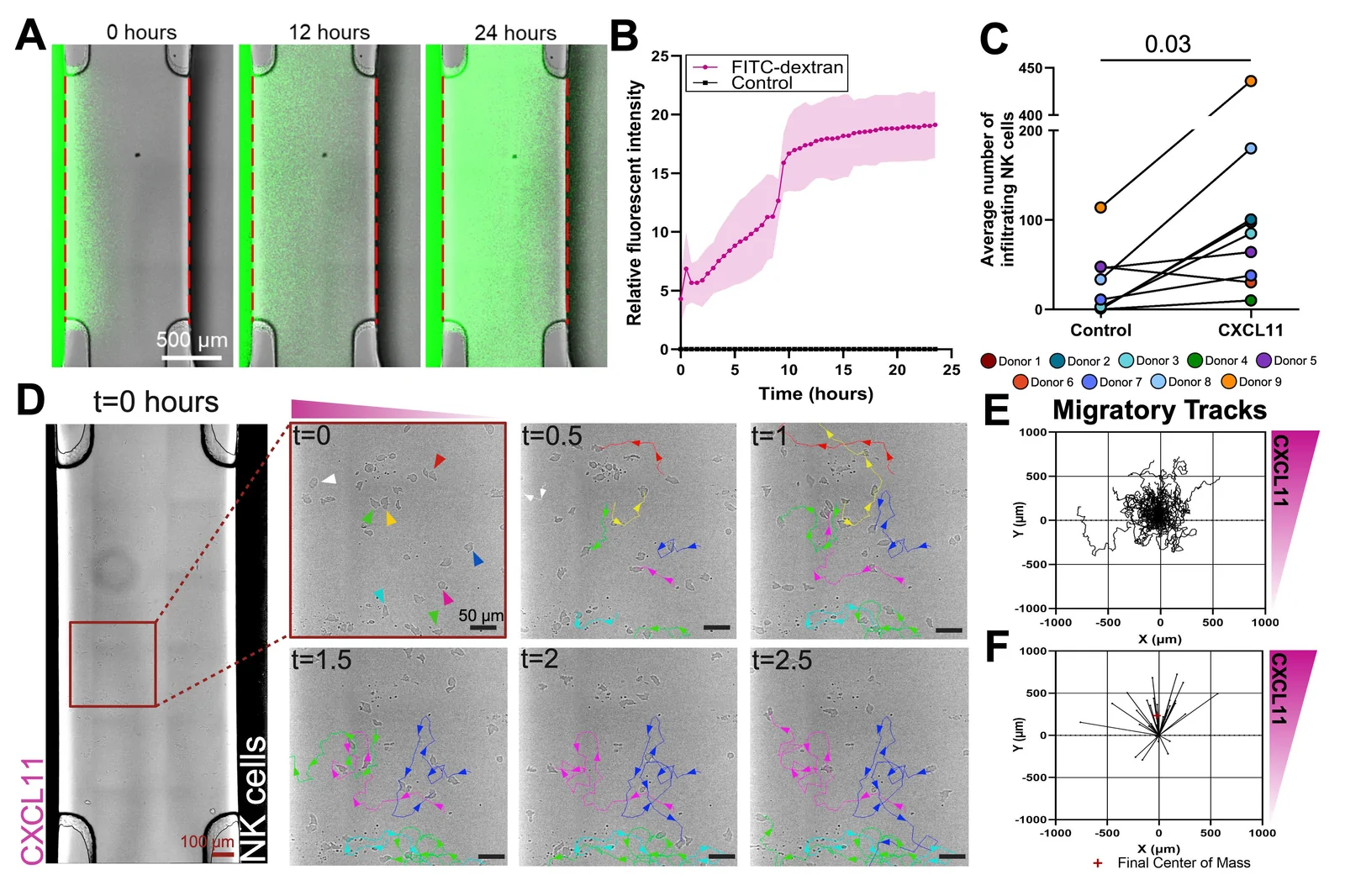
Directed NK cell migration in the 3D hydrogel. **(A)** Representative images of the diffusion of FITC-dextran (10 kDa) from the left chamber. The red dashed lines indicate the border of the observation area. **(B)** Quantification of the fluorescent intensity of the FITC-dextran gradient in the hydrogel relative to a control chamber that did not receive FITC-dextran. **(C)** Quantification of the average number of NK cells within the hydrogel after 5 days in culture in chambers with CXCL11 or medium only (control) (N = 2–3 chambers per donor). Statistical significance was determined using a paired Student’s t-test. **(D)** Annotated images of NK cells migrating through the hydrogel over 2.5 hours towards CXCL11. **(E)** Representative tracks and **(F)** endpoints only of primary NK cells in the chamber migrating towards CXCL11.

While the µ-Slide Chemotaxis is designed to maintain a stable, linear gradient, we confirmed the stability and profile of the chemoattractant gradient upon incorporation of the hydrogel using 10 kDa FITClabeled dextran to represent the diffusion of CXCL11 in chemotaxis chips containing degradable, adhesive hydrogels. The gradient diffusion was evaluated over 24 hours and verified by fluorescent microscopy (**Figure 2A**). Within 12 hours, signal from the FITC-dextran was observed in the hydrogel. At 24 hours, the FITC-dextran signal stops increasing, indicating there is no longer a gradient across the observation area (**Figure 2B**). Since the chemokine gradient is lost after 24 hours, changing the media in both chambers and refreshing the chemokine daily was required to re-establish a chemokine gradient for long-term migration studies. To determine if CXCL11 transport was affected by the hydrogel composition, we evaluated release of CXCL11 from various hydrogel formulations after 24 hours (**Supplementary Figure S3**). Hydrogels functionalized with MMP degradable peptide sequences, adhesion peptides, and/or HA demonstrated reduced release of CXCL11 compared to non-functionalized hydrogels, suggesting potential interactions between these biological moieties and the chemokine. However, the release was comparable across the functionalized hydrogels used to study NK cell migration.

### 4.3 NK cell migration towards a chemoattractant

Directed migration in the chemotaxis chip was evaluated by comparing NK cell migration towards CXCL11 or a media control. After primary cell isolation, the degradable, adhesive hydrogel was crosslinked in the observation area and rinsed in PBS; IL-2-containing media with CXCL11 was added to one reservoir and NK cells (4x10^6^ cells/mL) in IL-2-containing media were added to the opposite reservoir. The media and CXCL11 were refreshed daily, except in the control condition which received only media (without CXCL11) daily. After 2 days in the CXCL11-containing chemotaxis chip, NK cells were present in the hydrogel and cell numbers continued to increase over time in culture. With the exception of donor 6, CXCL11 was necessary for the cells to migrate into the hydrogel, as evidenced by greater NK cell accumulation in the hydrogels compared to media controls (**Figure 2C**). In general, higher CXCR3 expression correlated with increased infiltration (**Supplementary Figure S10**). Given the donor-to-donor variability in migration behavior, the potential influence of demographic characteristics was examined. NK cell infiltration efficiency was compared with donor age, sex, and ABO blood type (**Supplementary Figure S11**). No clear associations were observed, although the analysis was limited by the small number of donors. These results suggest that other factors, such as NK cell subset composition or activation state, may contribute to the observed variability.

In this system, individual NK cells could be tracked over time to evaluate migratory parameters (**Figures 2D–F**; **Video S1** and **Video S2**), and 3D migration was confirmed by the presence of cells at different heights in the chamber (**Supplementary Figure S12**). Of note, only a small fraction of NK cells (less than 1%) infiltrated the hydrogel.

### 4.4 NK cells’ reliance on mesenchymal migration in 3D

**Figure 3:**
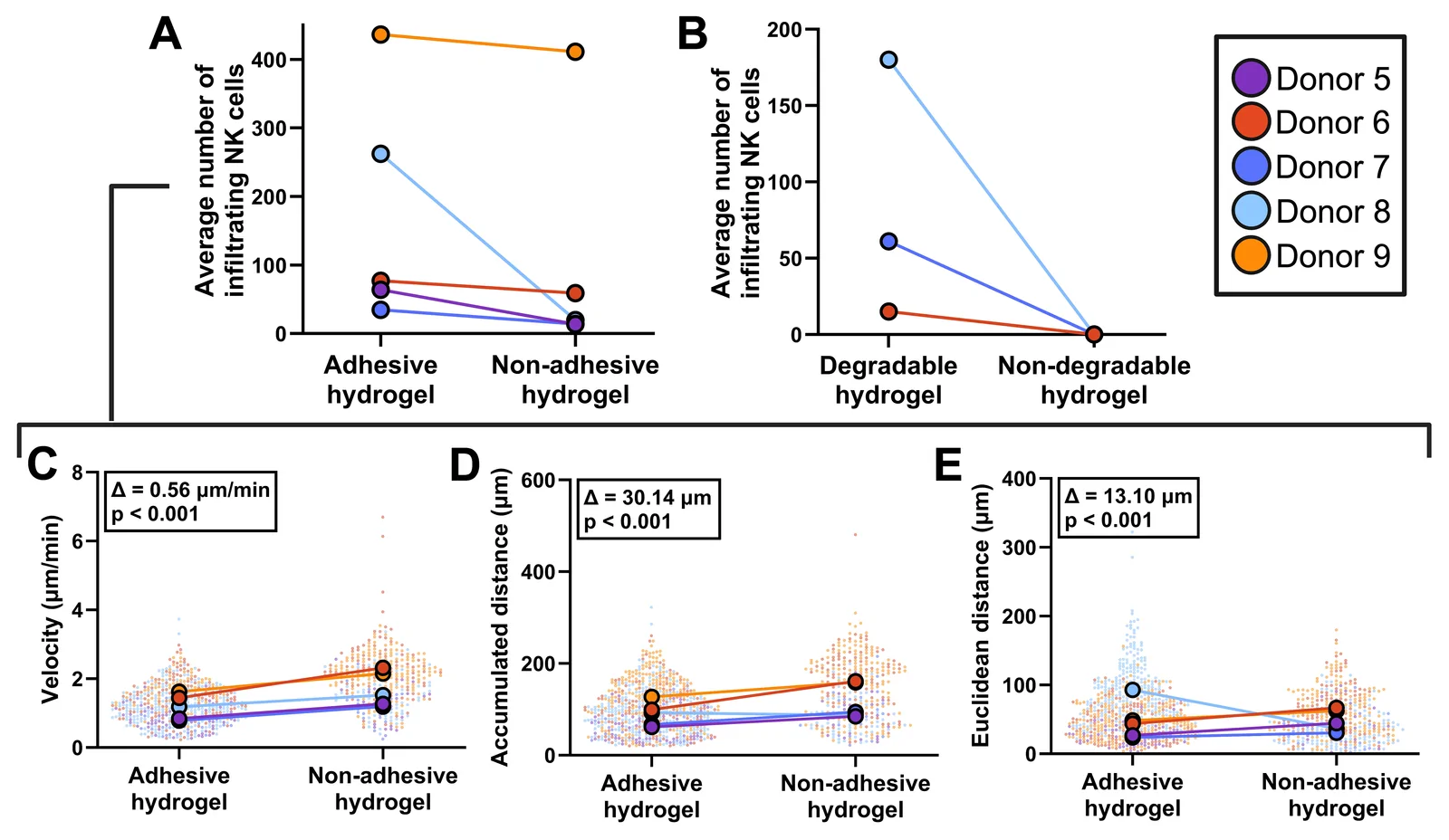
Evaluation of integrin and protease dependence of NK cells within 3D matrices. **(A)** Quantification of the average number of NK cells within the hydrogel after 5 days in culture in chambers with CXCL11 and either adhesive or non-adhesive hydrogels (N = 2–3 chambers per donor). **(B)** Quantification of the average number of NK cells within the hydrogel after 5 days in culture in chambers with CXCL11 and either degradable or non-degradable hydrogels (N = 2–3 chambers per donor). Quantification of individual cells migration parameters, including **(C)** velocity (µm/min), **(D)** accumulated distance (µm), and **(E)** Euclidean distance µm). Data are shown as scatter dot plots where small circles indicate individual cells, and large circles represent the corresponding donor mean. Statistical significance was determined using a linear mixed-effects model with experimental group as a fixed effect and donor as a random intercept. Effect sizes are reported as ∆ with corresponding p values where p *<* 0.05 was considered statistically significant.

Next, we investigated whether NK cells rely on mesenchymal migration mechanisms by evaluating their movement in hydrogels lacking either enzymatically degradable sites (PEG-RGD) or adhesion sites (PEG-dMMP-RAD). Fewer NK cells infiltrated these modified hydrogels compared to control hydrogels containing both adhesion and degradable sites, indicating that matrix degradation and adhesion are required to initiate migration (**Figures 3A, B**). Subsequently, we imaged the observation chambers of the chemotaxis chip for 2 hours with a Leica TCS SP8 confocal microscope, acquiring images every 2 minutes, and quantified the dynamic movement of cells within these matrices. Three key metrics were used to analyze NK cell movement: (1) velocity, which refers to the rate of change of the cell’s position over time, (2) accumulated distance, which measures the total path length traveled by a cell over a given period, regardless of direction, and (3) Euclidean distance, which is the straight-line distance between two points. Since no cells infiltrated the non-degradable hydrogel, further migration parameters were not evaluated in this group.

Migration parameters were analyzed using linear mixed-effects models with donor included as a random intercept to account for the hierarchical structure of the data (multiple cells per donor). Within non-adhesive hydrogels, NK cells migrated faster (1.4-fold) and traveled farther, with a 1.3- and 1.2-fold increase in accumulated and Euclidean distance, respectively, compared to cells in adhesive hydrogels (**Figures 3C–E**). NK cells from different donors showed variable reliance on integrin-dependent migration, with donors 6 and 9 exhibiting the strongest dependence on adhesion-mediated movement (**Figures 3C–E**). The average NK cell velocity in our PEG-based hydrogels ranged from 0.4-2.4 µm/min.

**Figure 4:**
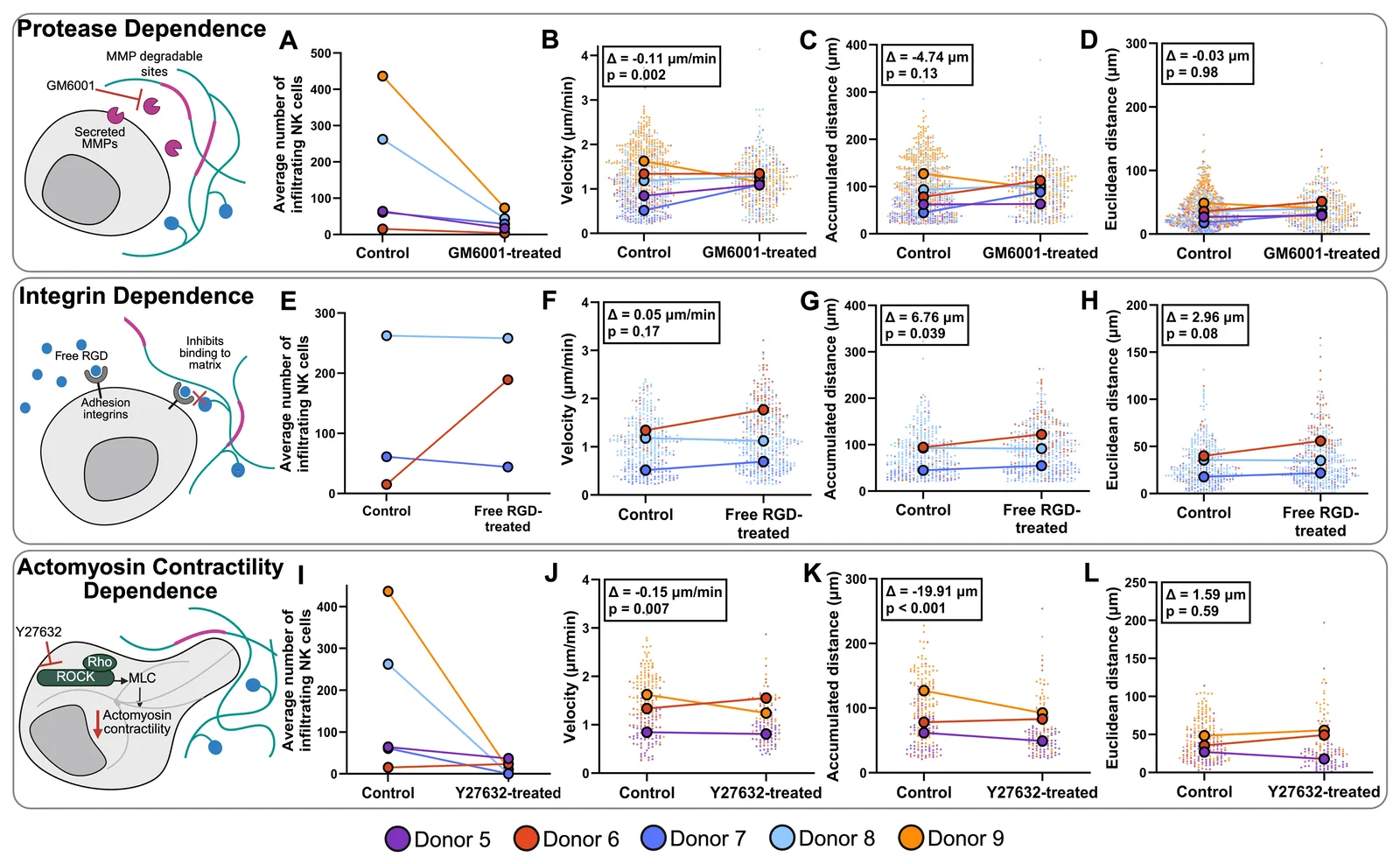
Characterizing NK cells mode of migration. **(A)** Evaluation of MMP-dependence in NK cell migration via inhibiting MMPs with GM6001 and quantifying the average number of NK cells that infiltrated the degradable, adhesive hydrogel (N = 2–3 chambers per donor). Quantification of individual cells migration parameters, including **(B)** velocity (µm/min), **(C)** accumulated distance (µm), and **(D)** Euclidean distance (µm). **(E)** Evaluation of integrin-dependence in NK cell migration via blocking integrin adhesion to PEG-RGD using soluble/free RGD and quantifying the average number of NK cells that infiltrated the degradable, adhesive hydrogel (N = 2–3 chambers per donor). Quantification of individual cells migration parameters, including **(F)** velocity (µm/min), **(G)** accumulated distance (µm), and **(H)** Euclidean distance (µm). **(I)** Evaluation of ROCK-dependence in NK cell migration via inhibiting ROCK with Y27632 and quantifying the average number of NK cells that infiltrated the degradable, adhesive hydrogel (N = 2–3 chambers per donor). Quantification of individual cells migration parameters, including **(J)** velocity (µm/min), **(K)** accumulated distance (µm), and **(L)** Euclidean distance (µm).Data are shown as scatter dot plots where small circles indicate individual cells, and large circles represent the corresponding donor mean. Statistical significance was determined using a linear mixed-effects model with experimental group as a fixed effect and donor as a random intercept. Effect sizes are reported as ∆ with corresponding p values where p *<* 0.05 was considered statistically significant.

Protease dependence was further evaluated by treating NK cells with 5 µM GM6001, a broad-spectrum MMP inhibitor, for two hours prior to and throughout the migration assay. Across all donors examined, fewer GM6001-treated NK cells infiltrated the hydrogel compared to control cells, although this reduction did not reach statistical significance (**Figure 4A**). Notably, the GM6001-treated NK cells that successfully invaded the hydrogel migrated 23% faster than control cells (**Figures 4B–D**). Substantial donor-to-donor variability was observed, with protease inhibition enhancing migration parameters in donor 7, reducing them in donor 9, and having minimal effects in other donors.

To examine the role of cell–matrix adhesion, integrins were blocked by pre-treating primary NK cells with 50 µM soluble RGD for two hours prior to and during the migration assay in adhesive, degradable hydrogels. Integrin blockade did not impair NK cell migration within this chemotaxis chip (**Figures 4E–H**) and resulted in substantial donor-to-donor variability (**Figure 4E**).

### 4.5 Donor-dependent variation in ROCK-mediated NK cell migration

While proteases and integrins are typically associated with mesenchymal migration, amoeboid-like migration relies primarily on actomyosin contractility and cytoskeletal dynamics, enabling cells to deform and move through confined spaces without proteolysis or strong adhesion. To assess the contribution of amoeboid migration to NK cell invasion, ROCK was inhibited by treating primary NK cells with 20 µM Y27632 for two hours prior to and throughout migration in adhesive, degradable hydrogels. ROCK inhibition reduced NK cell infiltration into the hydrogel and significantly decreased migration velocity and accumulated distance among invading cells (**Figures 4I–L**). In contrast, Euclidean distance was not significantly altered (**Figure 4L**). Dynamic migration parameters were evaluated only for donors 5, 6, and 9, as no treated cells were detected in the hydrogel for donors 7 and 8.

### 4.6 Hyaluronic acid enhances NK cell migration

To demonstrate the utility of the hydrogel-loaded chemotaxis chip, we incorporated tumor-relevant ECM components and explored their effects on NK cell migration. Solid tumors have higher hyaluronic acid (HA) content than healthy tissues (29, 30), which is associated with tumor growth and aggressiveness. Tumors contain both high molecular weight (HMW) and low molecular weight (LMW) HA, the latter resulting from the degradation of HMW HA by hyaluronidase produced by cancer and stromal cells (30). HMW HA has anti-inflammatory, anti-proliferative, and antiangiogenic effects (94), inhibiting tumor growth (95) and reducing the production of MMPs (96), while LMW HA promotes inflammation, proliferation, angiogenesis (94), and metastasis (97). Furthermore, HA is thought to influence immune cell migration (31, 32), yet the influence of HA on NK cell migration parameters has not been studied. Given the limited understanding of the influence of the different molecular weights of HA on NK cell migration, we set out to investigate this relationship in the established chemotaxis chip.

**Figure 5:**
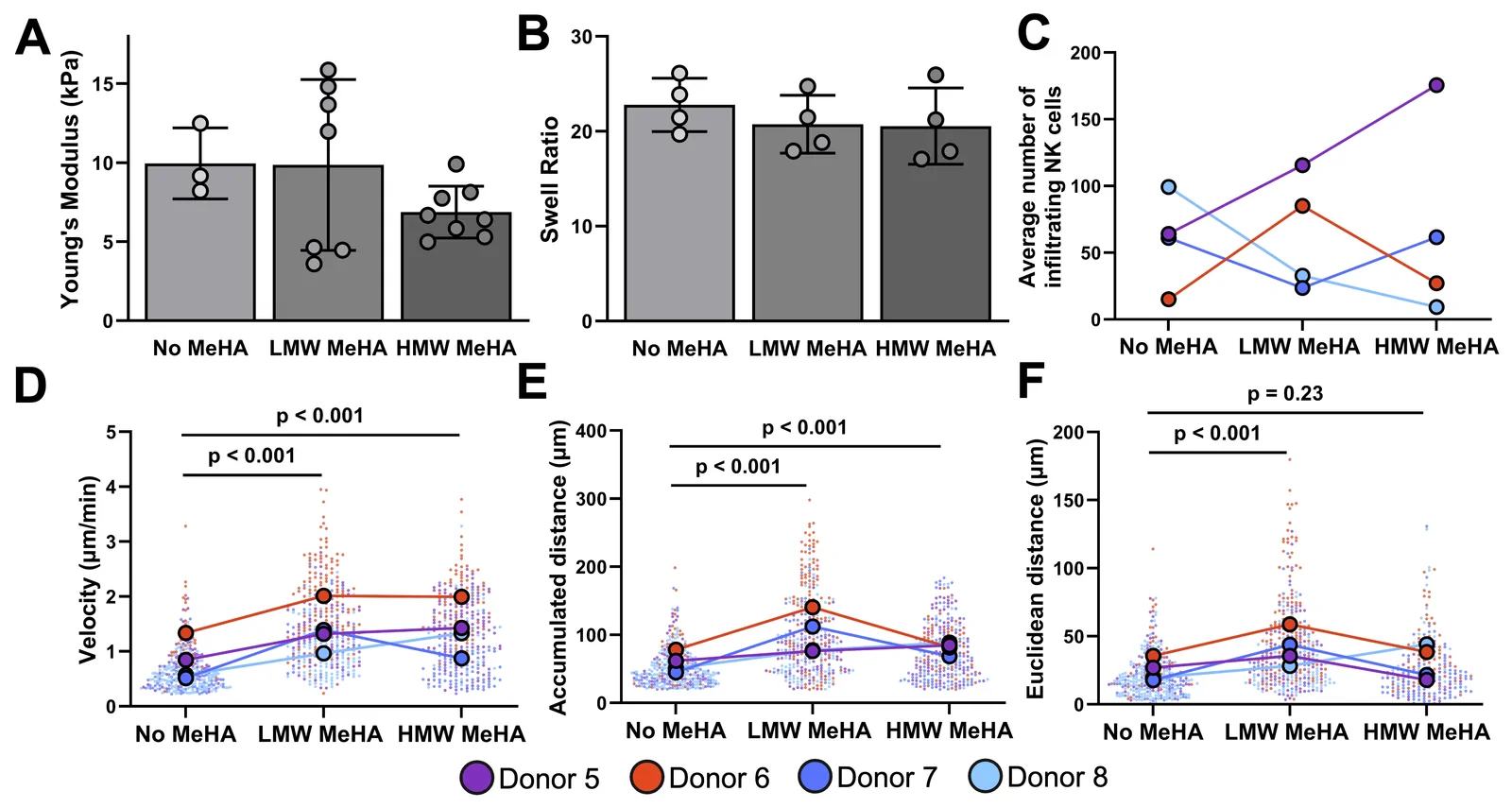
Effect of HA on NK cell migration. Either 500µg/mL of HMW MeHA, LMW MeHA, or no MeHA were added to degradable, adhesive hydrogels without significantly altering the **(A)** Young’s modulus or **(B)** swell ratio. **(C)** Quantification of the average number of NK cells within the hydrogel after 5 days in culture in chambers with CXCL11 and hydrogels containing MeHA. Quantification of individual cells migration parameters, including **(D)** velocity (µm/min), **(E)** accumulated distance (µm), and **(F)** Euclidean distance (µm). Data are shown as scatter dot plots where small circles indicate individual cells, and large circles represent the corresponding donor mean. Statistical significance was determined using a linear mixed-effects model with experimental group as a fixed effect and donor as a random intercept. P values where p < 0.05 was considered statistically significant.

HMW HA and LMW HA were chemically methacrylated (MeHA) and incorporated into degradable, adhesive hydrogels without altering their mechanical or physical properties, as confirmed by comparable Young’s modulus and swelling ratios (**Figures 5A, B**). Inclusion of MeHA did not result in a consistent overall change in NK cell numbers, and substantial donor-to-donor variability was observed, with HA increasing infiltration in some donors and decreasing it in others (**Figure 5C**). However, NK cell migratory behavior was enhanced in HA-containing hydrogels, as cells migrated 1.8-fold faster and traveled 1.7- or 1.4-fold greater accumulated distances in matrices containing LMW MeHA or HMW MeHA, respectively (**Figures 5D, E**). Donor-specific differences were observed in NK cell migration within hydrogels containing LMW or HMW MeHA (**Figures 5C–F**). NK cells from donors 5–7 exhibited greater migration distance and speed in LMW MeHA, whereas NK cells from donor 8 migrated farther and faster in HMW MeHA relative to hydrogels without MeHA.

## 5 Discussion

Understanding and optimizing NK cell migration is essential for improving their effectiveness in solid tumors, ensuring that these immune cells can efficiently locate, engage, and destroy cancerous tissue. However, conventional functional assays for NK cells typically do not include 3D migration. The chemotaxis chip presented here introduces a platform to investigate human NK cell migration mechanisms within an engineered 3D hydrogel, where the biochemical properties can be independently controlled, offering a multi-parametric readout of infiltration, velocity, and distance.

### 5.1 Methodological advancements

In developing this platform, we established a method to polymerize hydrogel formulations with well-defined mechanical and biochemical properties directly within the chemotaxis chip. The resulting mechanical properties were in the reported range for various solid tumors, which can range from 4–186 kPa depending on the tumor type and location (98). For example, breast cancer lesions have reported elastic moduli of 10–42 kPa, while healthy breast tissue has reported moduli of approximately 3 kPa (99). Although the present system is not designed to model a specific tumor type, the stiffness is physiologically relevant and consistent with many solid tumor microenvironments. Within this matrix architecture, our method allows us to sustain a functional chemotactic gradient relevant for primary NK cell migration. Our finding that CXCR3 was the most prevalent receptor on primary NK cells is highly consistent with other studies reporting the predominant expression of CXCR3 in primary NK cells compared to other chemokine receptors (20, 100), which may be a consequence of the IL-2 stimulation used (24). This receptor prevalence justified the selection of CXCL11 as our target chemoattractant, given its status as the most potent ligand for CXCR3 (93). Ultimately, this setup provides a method for characterizing NK cell motility and migration within the hydrogel, offering a direct way to evaluate how these physical and biochemical cues drive cell motility.

### 5.2 Mechanistic evaluation of NK cell motility

Testing primary human NK cells within this system revealed distinct migration profiles and behavioral adaptations across different hydrogel environments. When tracking cellular infiltration, the observation that less than 1% of NK cells penetrated the matrix reflects the known inefficiency of immune penetration into dense environments. This is consistent with solid tumor observations, such as pancreatic ductal adenocarcinoma, where NK cells comprise less than 0.5% of tumor-infiltrating immune cells (101). While this limited infiltration profile might naturally enrich for highly migratory subsets, our demographic analysis (evaluating age, sex, and ABO blood type) revealed no clear structural associations, though it was limited by the small number of donors. This suggests that other baseline factors, such as specific NK cell subset compositions or activation states, drive the observed donor-to-donor variability in matrix entry.

Interestingly, the average NK cell velocity across our PEG-based hydrogels (0.4–2.4 µm/min) was slower than velocities reported in other 3D systems, such as collagen [3 µm/min in 3.0 mg/mL (51); 6.5–8 µm/min in 1.2 mg/mL (56)], Matrigel [3.6–5.4 µm/min (60)], or carbomer hydrogels [5.8–6.5 µm/min (56)]. These slower velocities may be attributed to matrix structural properties like hydrogel mesh size, although differences in cell source, imaging parameters, and tracking methodologies can also influence measured speed. NK cell migration velocity is functionally important, as faster migration near bystander cells can reduce search time and enhance cytotoxic efficiency. While a direct correlation between velocity and cytotoxicity has not yet been established, parallels with T cell behavior suggest that such relationships are worth exploring in future studies (102).

Mechanistically, our data indicate that while protease-dependent matrix degradation and integrin engagement are heavily leveraged to initiate infiltration, their presence subsequently influences dynamic movement. For instance, cells within non-adhesive hydrogels or under broad MMP inhibition via GM6001 exhibited restricted initial entry, yet the subset that successfully invaded migrated 23% faster. Although the GM6001 concentration was selected based on prior literature (83), incomplete inhibition cannot be excluded, or alternative proteases may contribute to hydrogel degradation. Notably, our MMPdegradable peptide sequence has also been reported to be cleaved by plasmin (103), which NK cells produce (104). Taken together, these data suggest that MMP inhibition reduces overall infiltration but does not fully abrogate migration; cells that do infiltrate retain the ability to migrate at comparable or greater velocities, potentially by engaging alternative, protease-independent mechanisms such as amoeboid-like migration.

This behavioral adaptability is further supported by our integrin experiments. While replacing the RGD sequence with non-binding RAD peptide restricted infiltration, competitive inhibition with soluble RGD did not impair motility, showing that NK cells maintain migration capacity even when adhesion pathways are only partially blocked. Integrin-independent migration in 3D environments has also been reported in other immune cell types. For example, dendritic cells require integrin-mediated adhesion for 2D migration but not for 3D migration in natural hydrogels (40, 105). T cells physically adhere while migrating through 3D collagen matrices, where contact guidance directs forward migration without overt matrix degradation (48), and macrophages can switch migration modes depending on ECM characteristics (37). Conversely, our results with ROCK inhibition using Y27632 show a significant drop in velocity and accumulated distance among invading cells. This demonstrates that amoeboid-like elements of their motility rely structurally on actomyosin contractility and cytoskeletal dynamics to deform and move through confined spaces without strong adhesion. Collectively, these findings support the concept that NK cells adapt their migratory strategy according to ECM composition, adhesion ligand availability, and receptor engagement, underscoring the plasticity of immune cell trafficking within 3D microenvironments.

Though the number of infiltrating NK cells varied among donors, their motility was consistently elevated in HA-bound matrices. Specifically, the 1.8-fold increase in velocity and longer tracking distances align with evidence that glycosaminoglycan integration optimizes the structural matrix for immune cell motility. This enhanced migration matches trends observed in other adoptive immunotherapies; for example, HA-rich environments support the functional proliferation and trafficking of NK92 cells (106), and HMW matrices can boost T-cell migration velocity compared to HA-free controls 62). The distinct donor-to-donor divergence in preference for LMW versus HMW matrices is an interesting finding. Because immune cells navigate HA architectures using specific surface proteins, particularly transmembrane receptors like CD44 that regulate rolling adhesion and downstream migration cascades (107), screening baseline receptor levels on these primary cells will be an important step toward understanding these individual donor patterns.

The donor-to-donor variability observed across our integrin, ROCK, and HA assays underscores the personalized nature of primary NK cell migratory behavior. In this study, cells from healthy donors were predominantly composed of the CD56^dim^CD16^+^ cytotoxic subset, meaning our results directly reflect the population most relevant for antitumor activity. This suggests that the chemotaxis chip could serve as a valuable platform for functionally profiling patient-derived NK cells and tailoring immunotherapeutic strategies accordingly.

### 5.3 Advantages, limitations, and applications

A key advantage is the use of an engineered synthetic hydrogel over traditional natural matrices like collagen or Matrigel. By utilizing a modular PEG-based chemistry, researchers can adjust mechanical and biochemical properties independently. This degree of control allows the decoupling of matrix stiffness from biochemical factors, making it possible to study NK cell migratory behavior in a systematic, quantifiable manner that is difficult to achieve in poorly defined natural tissues. Furthermore, the fully synthetic nature of the matrix eliminates the lot-to-lot variability inherent to animal-derived gels, ensuring strict experimental consistency. The optical clarity of the PEG hydrogel also avoids the light-scattering issues common to fibrous matrices, directly facilitating high-resolution 3D live-imaging and automated cell tracking.

While our platform isolates and captures key aspects of NK cell migration mechanisms, it has inherent limitations as a simplified model. By design, it does not recapitulate the full cellular or structural complexity of the solid tumor microenvironment, such as stromal cell interactions, interstitial fluid pressure, or dense tumor-tissue boundaries. Additionally, while the system provides quantitative readouts of motility, a direct correlation between these migratory metrics and subsequent therapeutic function (e.g., *in vivo* tumor clearance) remains to be definitively established. Future iterations of this method incorporating larger patient cohorts will improve the generalization of these findings, and integrating parallel downstream assays evaluating cytotoxicity, degranulation, and cytokine release will be necessary to fully map how distinct migration profiles translate to immunotherapy efficacy.

In conclusion, the 3D chemotaxis chip establishes a robust, standardized methodology to polymerize biomimetic hydrogels within a microfluidic architecture, sustain functional chemokine gradients, and quantify 3D immune cell motility. This platform offers valuable utility for both basic immunology and translational medicine. Specifically, it can be applied to evaluate bioengineering strategies aimed at improving NK cell trafficking through relevant matrix barriers, or to interrogate the fundamental biological mechanisms governing immune cell responses to ECM cues. Moreover, in the context of adoptive cellular therapies, this method presents a scalable framework to screen patient-specific migratory behaviors during the manufacturing process, filtering for candidates with optimal infiltration potential. Beyond oncology, the modular nature of this hydrogel chip allows it to be readily adapted for mechanistic studies of other migration-dependent processes, including wound healing, transplant rejection, and placental development.

## Data Availability

The original contributions presented in the study are included in the article/**Supplementary Material**. Further inquiries can be directed to the corresponding author.
