# Supplementary material for: 3D chemotaxis chip for investigating natural killer cell migration mechanisms
Source: Front Immunol. 2026 Aug 13;17:1762203. doi: 10.3389/fimmu.2026.1762203 (PMC13518586; doi:10.3389/fimmu.2026.1762203)
Supplement: Supplementary file 1 [file DataSheet1.pdf]

Supplemental Material for 3D Chemotaxis Chip for Investigating NK Cell Migration

Madison Temples, Suzanne Lightsey, Tiffany Conklin, Rylee Newport, Edward Phelps, and Blanka Sharma

---

Figure S1: Graphical abstract …..………………………………………… Page 2

Table S1: NK cell donor demographics ………………………………… Page 3

Figure S2: Representative gaiting strategy ……………………………. Page 4

Figure S3: CXCL11 diffusion in hydrogels……………………………… Page 5

Figure S4: Functionalization of PEG with dMMP peptide …………..… Page 6

Figure S5: Functionalization of PEG with RGD peptide ……………... Page 7

Figure S6: HNMR analysis of LMW and HMW MeHA …..…………… Page 8

Table S2: Hydrogel precursor solutions ………………………………… Page 9

Figure S7: Viability of primary NK cells after inhibitor treatment …….. Page 10

Figure S8: Quantile–quantile (QQ) plots of residuals …………..…… Page 11

Figure S9: Characterization of primary NK cells ……………………… Page 12

Figure S10: Donor expression of CXCR3……………………………… Page 13

Figure S11: Donor demographics correlation…………………...…… Page 14

Figure S12: NK cell migration in 3D…………………………………….. Page 15

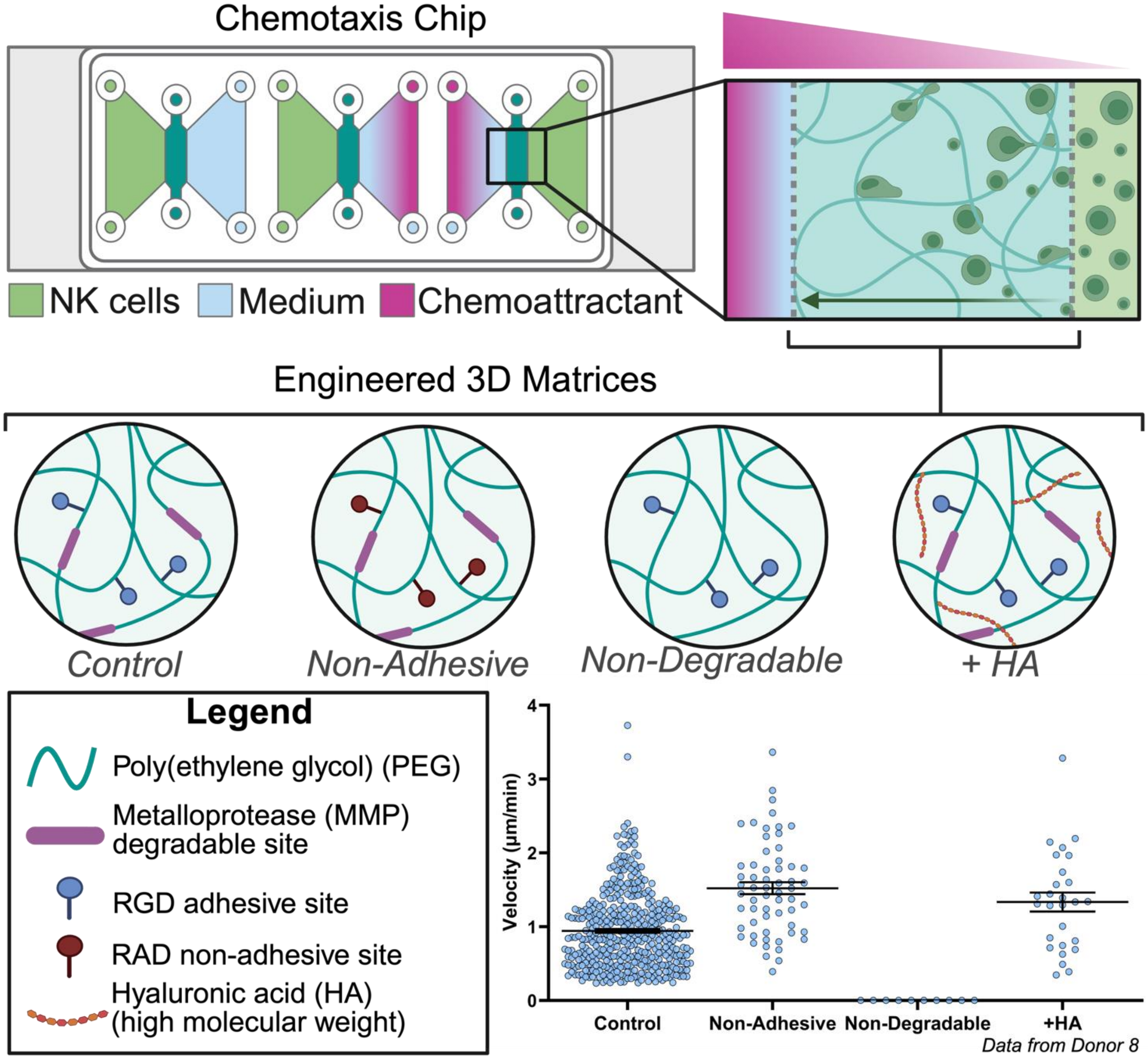

Figure S1. Graphical Abstract.

Donor number	Sex	Age	Ethnicity	ABO
1	Female	53	Caucasian	A-
2	Male	34	Caucasian	O+
3	Male	23	Caucasian	A+
4	Female	24	Hispanic	A-
5	Female	62	Caucasian	A+
6	Female	27	Caucasian	O+
7	Female	33	Caucasian	A+
8	Male	55	Caucasian	A+
9	Female	70	Caucasian	O+

Table S1. NK cell donor demographics.

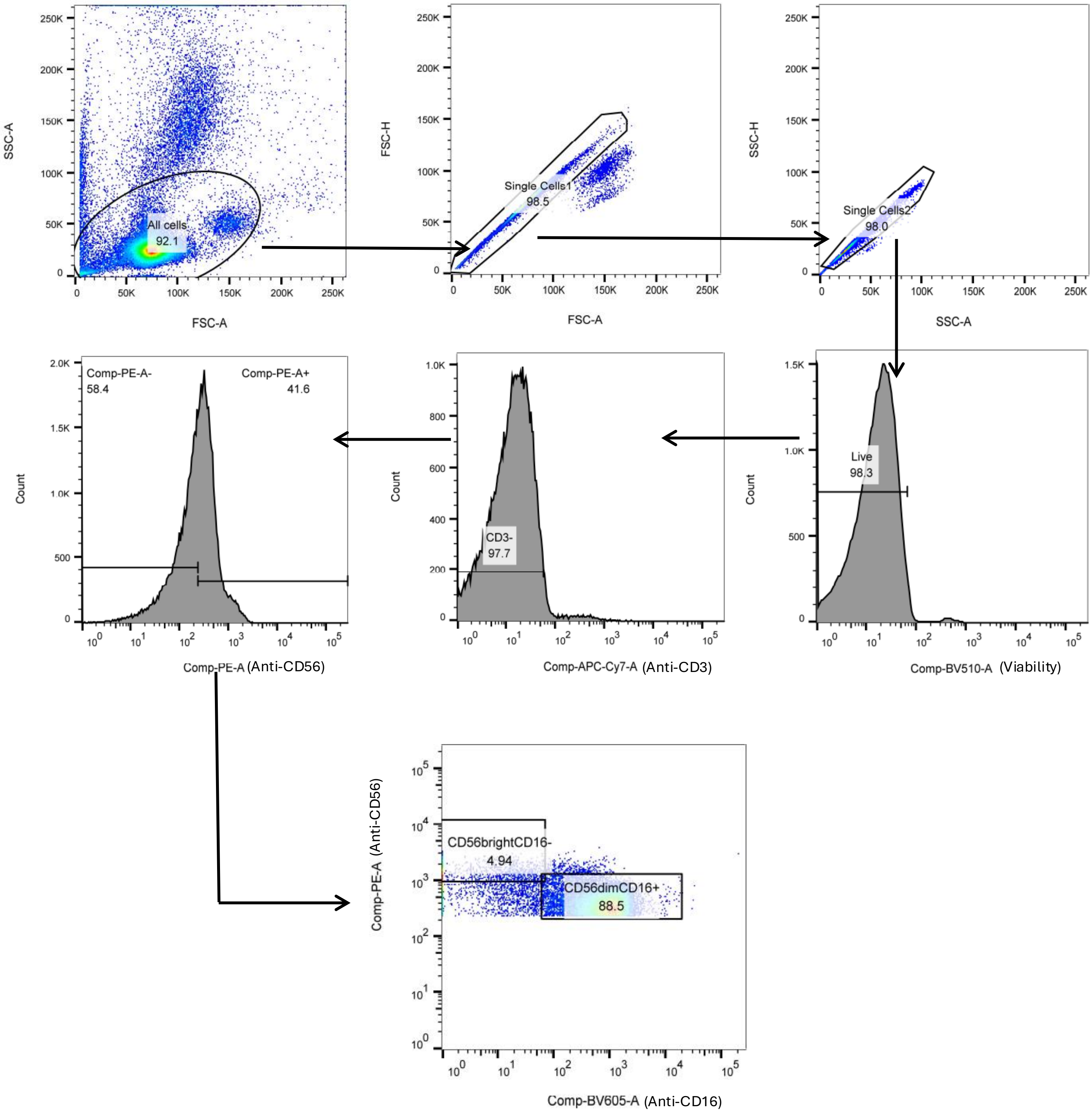

Figure S2. Representative gating strategy for isolated primary NK cells. After gating on single, viable cells, $CD3^+$ T cells were excluded to define the $CD3^-$ population. NK cells were identified as $CD56^+$ cells, and CD56 and CD16 expression were used to delineate NK cell subpopulations.

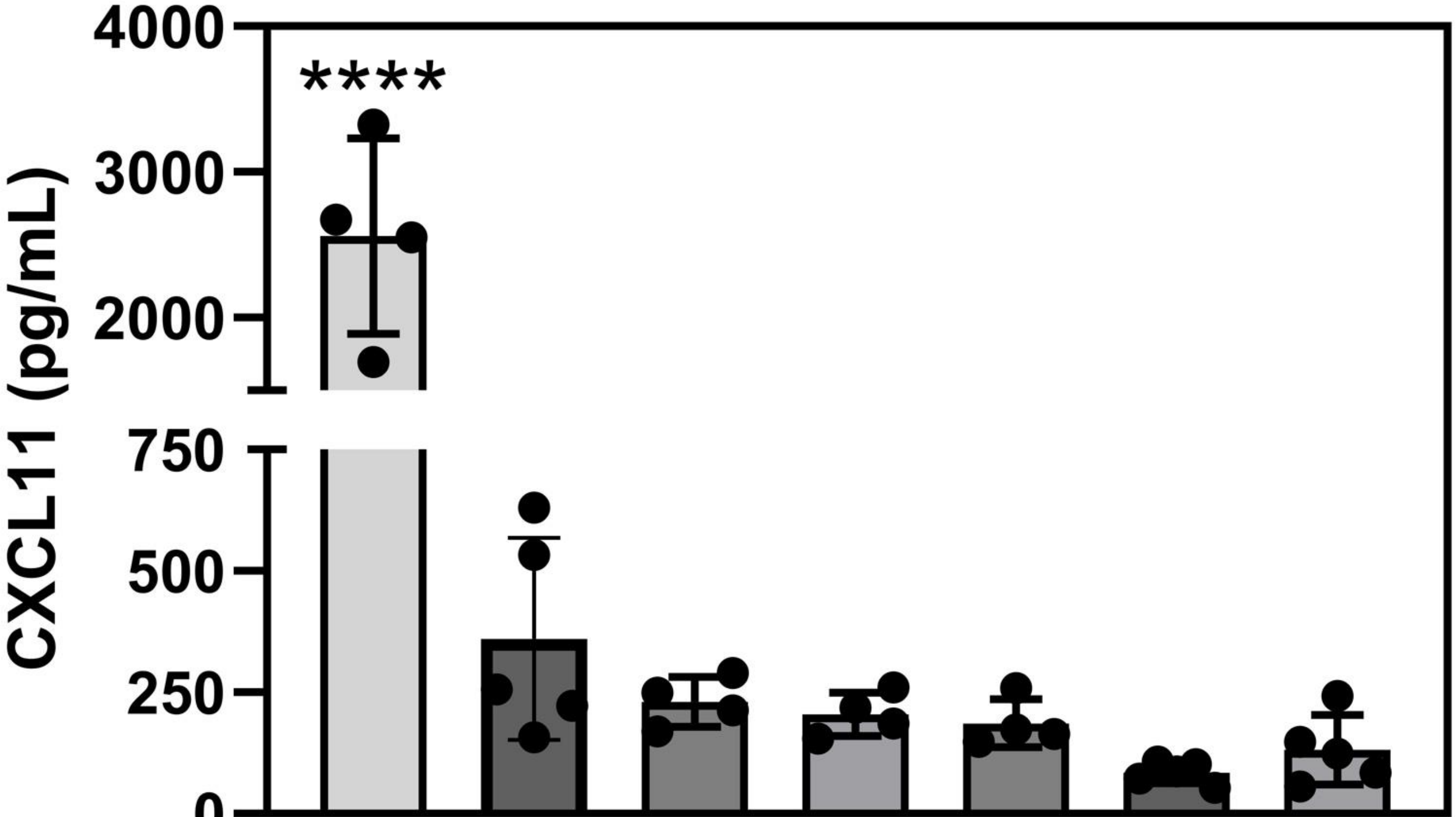

MMP degradable	-	-	+	+	+	+	+
RGD	-	+	-	+	-	-	-
RAD	-	-	-	-	+	-	-
LMW MeHA	-	-	-	-	-	+	-
HMW MeHA	-	-	-	-	-	-	+

Figure S3. CXCL11 diffusion in hydrogels with different functional sites. CXCL11 was encapsulated in hydrogels containing MMP-degradable sites, integrin motifs (5 mM), or MeHA (500 µg/mL) to assess differences in CXCL11 diffusion. Hydrogels with CXCL11 were incubated in PBS at 37 °C for 24 hours. The supernatant was collected to assess CXCL11 release, and CXCL11 concentration was quantified via ELISA (R&D Systems, Cat. #DCX110). Statistical significance was determined using a one-way ANOVA followed by Tukey's multiple comparisons test, where ****$p<0.0001$.

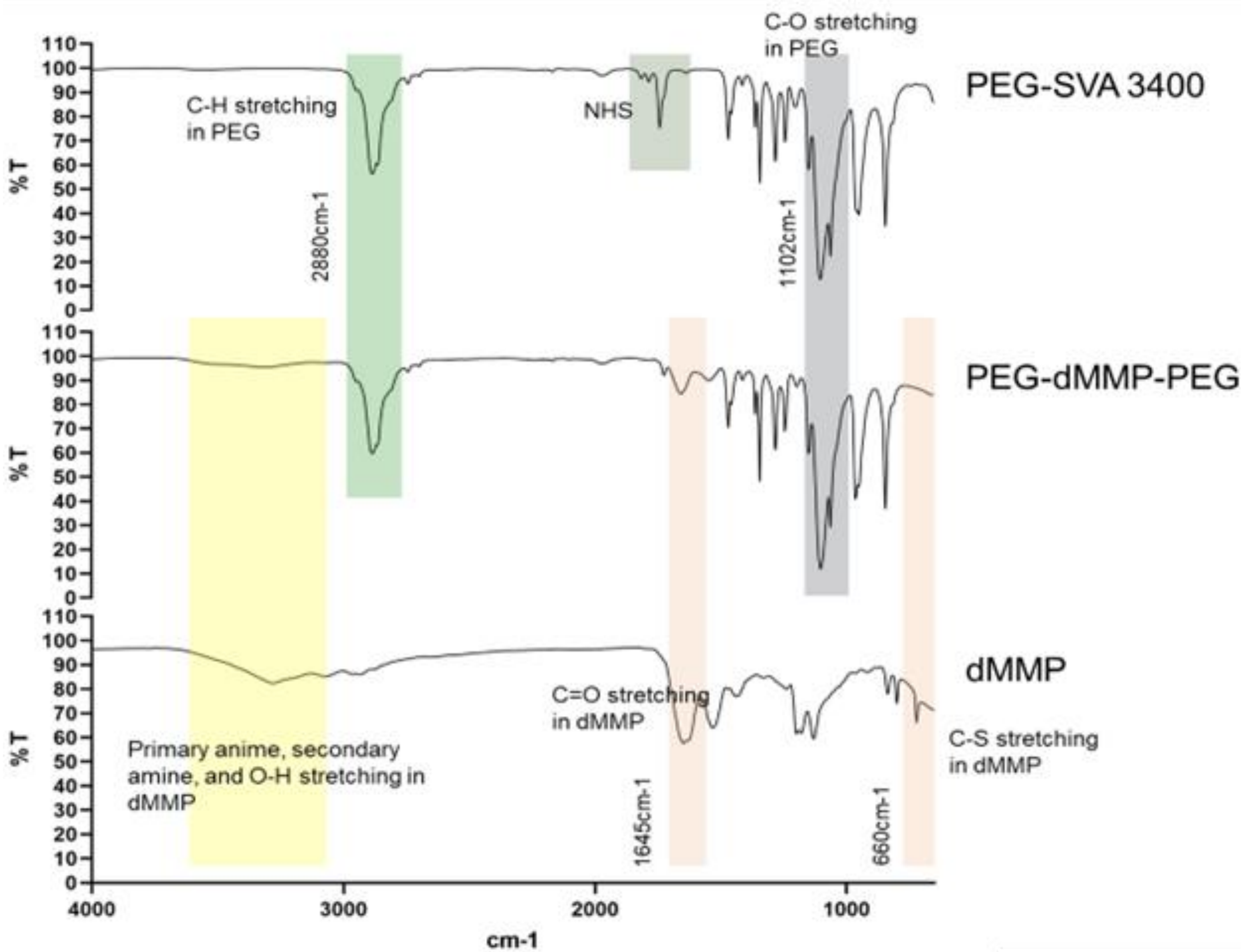

Figure S4. FTIR spectrum to confirm functionalization of PEG with dMMP peptide. Top is spectrum for acryl-PEG-SVA (MW 3400), middle is spectrum for PEG-dMMP-PEG, and bottom is spectrum for dMMP peptide.

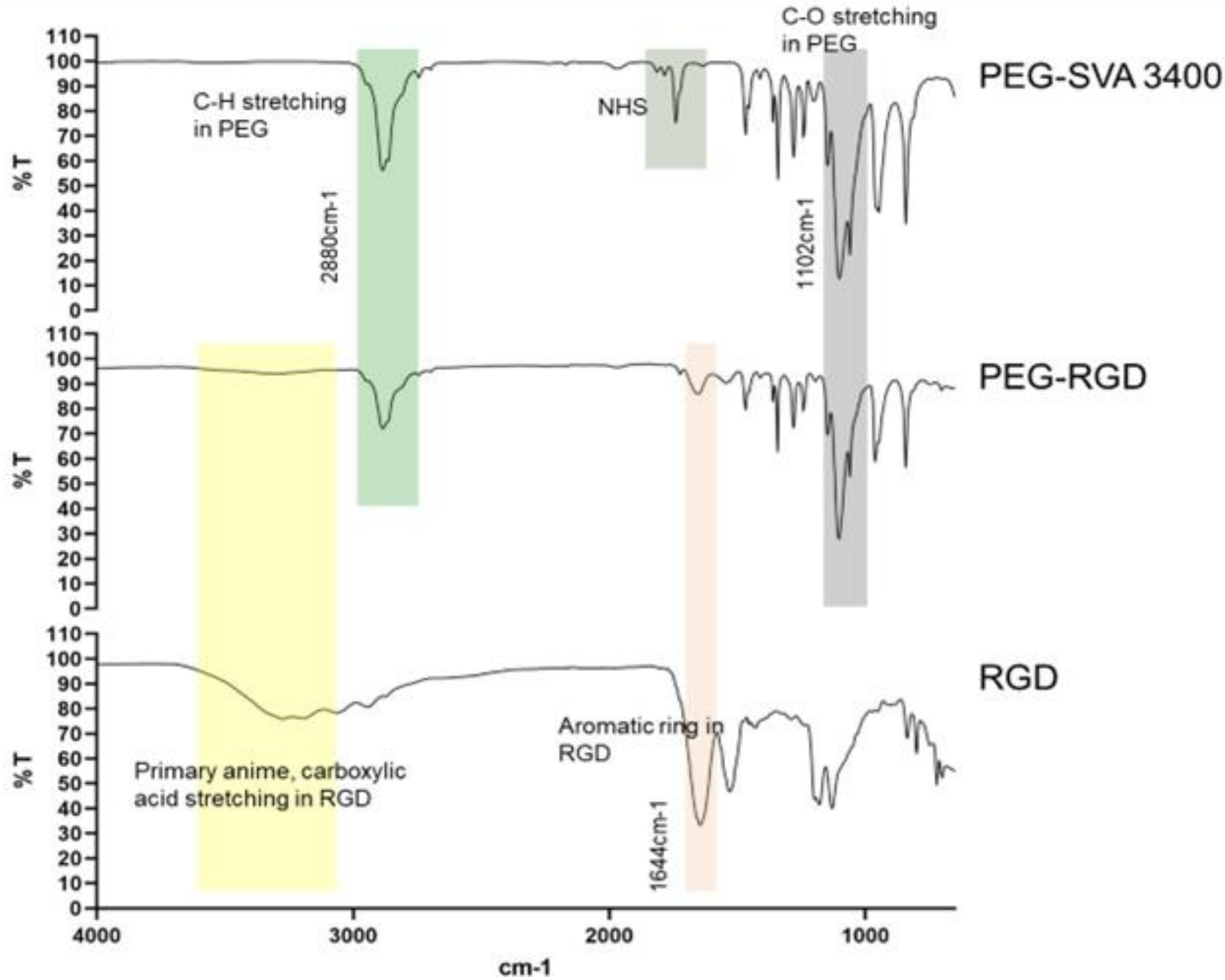

Figure S5. FTIR spectrum to confirm functionalization of PEG with RGD peptide. Top is spectrum for acryl-PEG-SVA (MW 3400), middle is spectrum for PEG-RGD, and bottom is spectrum for RGD peptide.

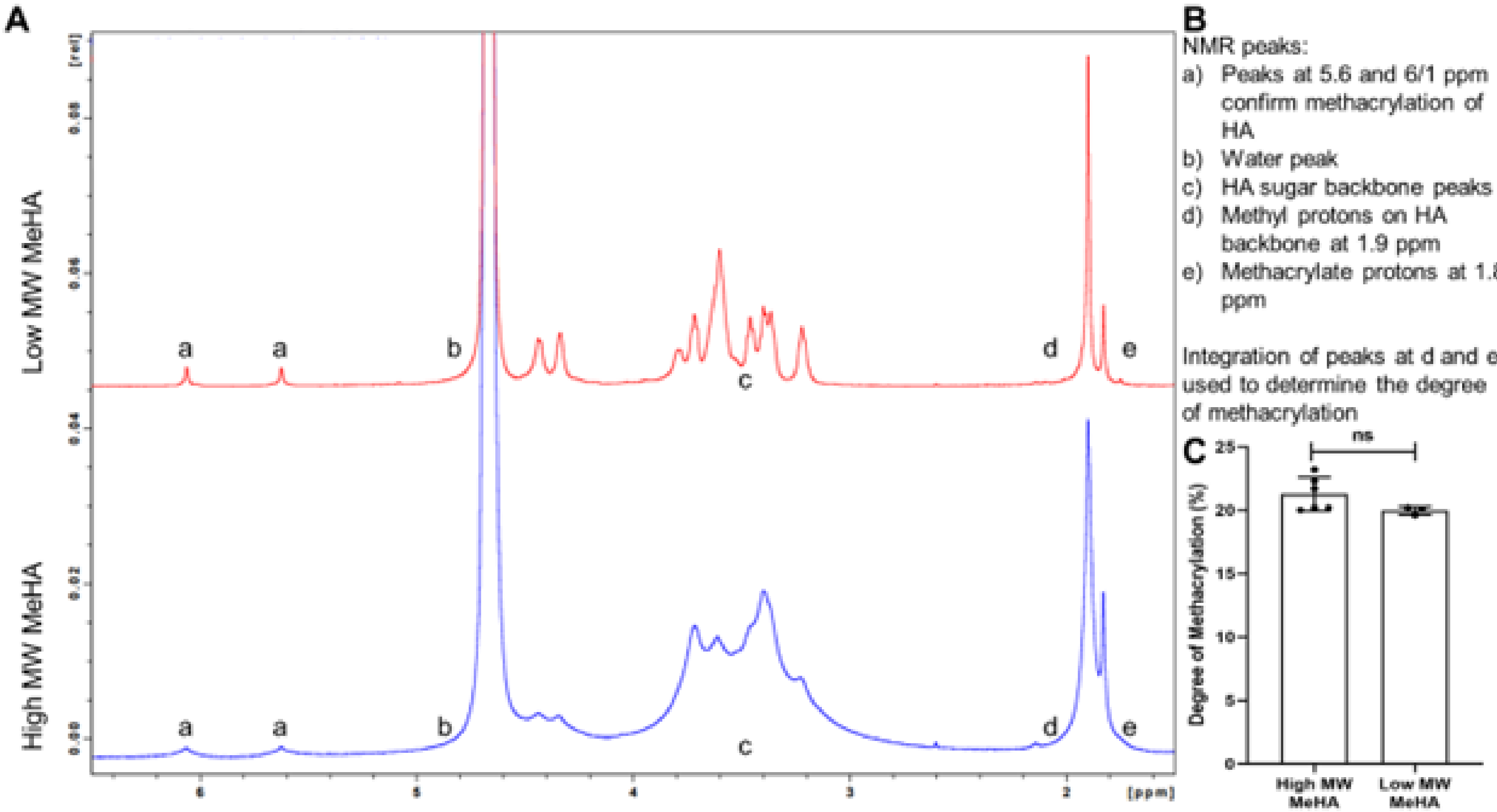

Figure S6. HNMR analysis of LMW and HMW MeHA. (A) Spectrums (pulse-acquired method) of LMW and HMW MeHA. (B) Key for the letter tags on the spectrums in A. (C) Quantification of the degree of methacrylation, based on the integration of peaks at d and e, for low and high MW MeHA.

Hydrogel name	Precursor Solution Composition
Degradable, adhesive hydrogel	10% (w/v) PEG-dMMP-PEG (8 kDa) and 5 mM PEG-RGD
Non-degradable, adhesive hydrogel	10% (w/v) PEG-PEG-PEG (10 kDa) and 5 mM PEG-RGD
Degradable, non-adhesive hydrogel	10% (w/v) PEG-dMMP-PEG (8 kDa) and 5 mM PEG-RAD
LMW MeHA	10% (w/v) PEG-dMMP-PEG (8 kDa), with 5 mM PEG-RGD, and 500 µg/mL LMW MeHA (10-30 kDa)
HMW MeHA	10% (w/v) PEG-dMMP-PEG (8 kDa), with 5 mM PEG-RGD, and 500 µg/mL HMW MeHA (750-1000 kDa)

Table S2. Hydrogel precursor solutions.

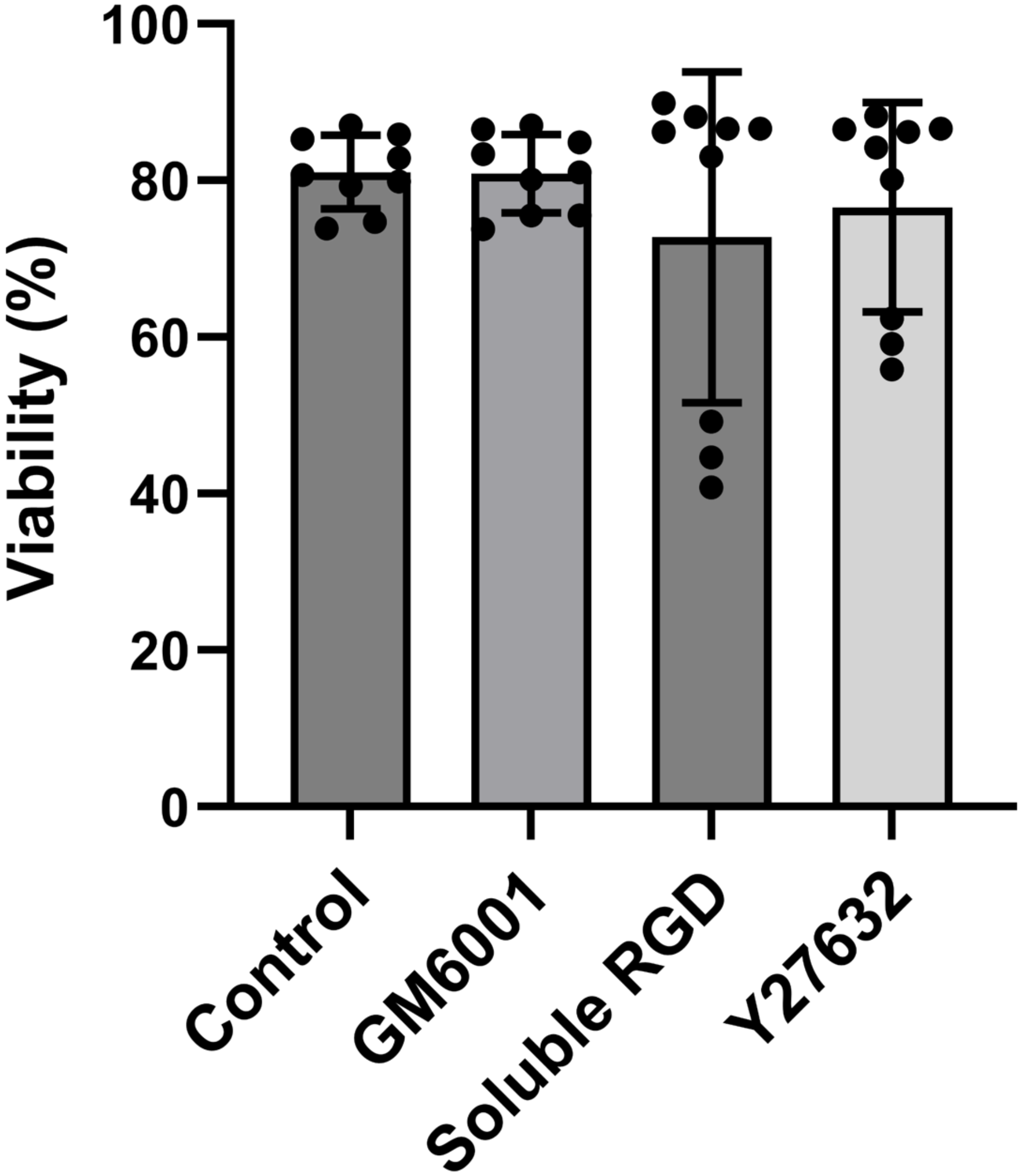

Figure S7. Viability of primary NK cells after inhibitor treatment. NK cells were treated with the inhibitors used in this study for 2 hours and the viability was determined by flow cytometry. Statistical significance was determined using a one-way ANOVA followed by a Tukey's multiple comparison test.

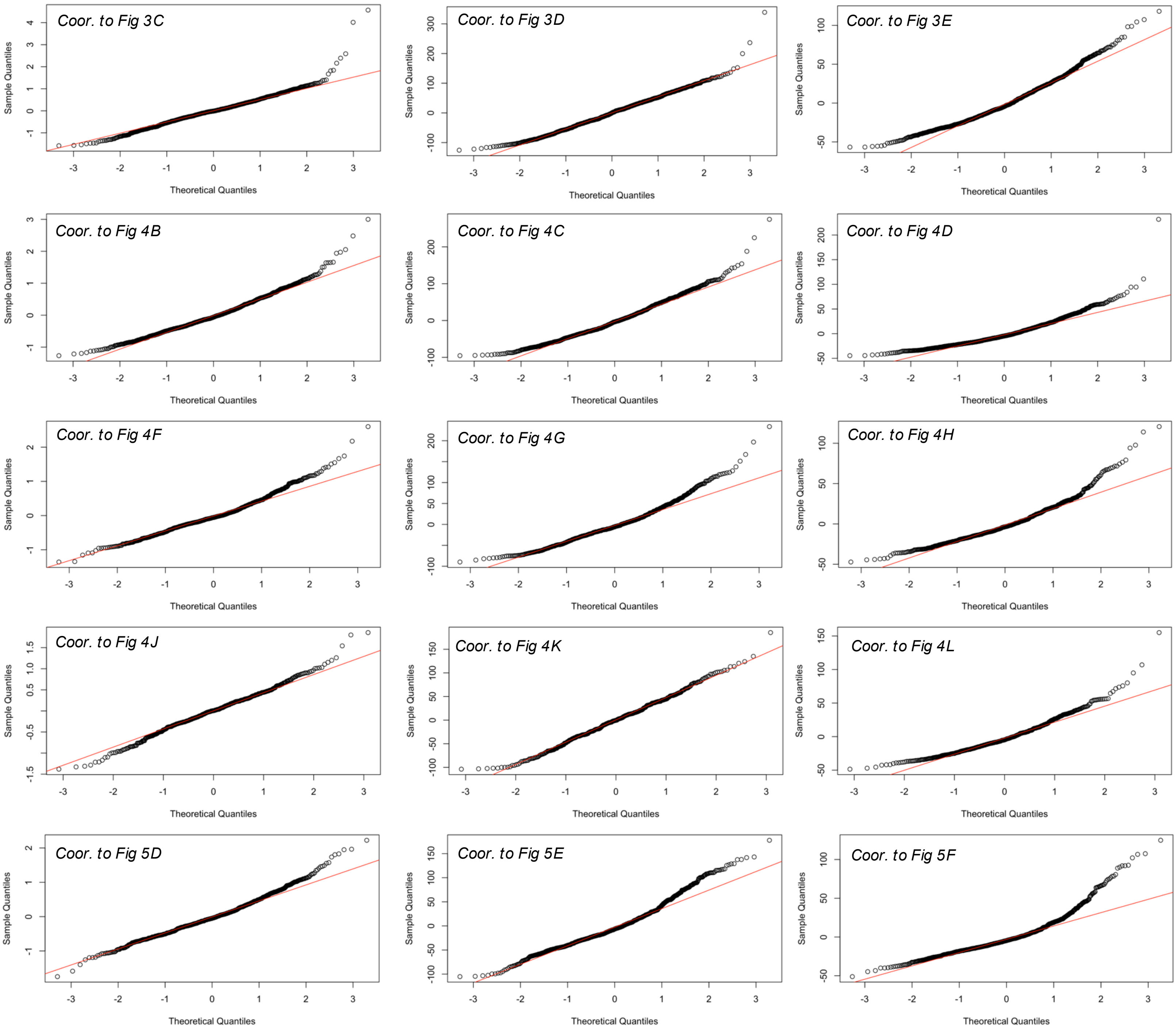

Figure S8. Quantile–quantile (QQ) plots of residuals from the linear mixed-effects model. Residuals are plotted against the theoretical quantiles of a normal distribution to assess the assumption of normality. Points closely following the diagonal reference line indicate that model residuals are approximately normally distributed, supporting the validity of model assumptions.

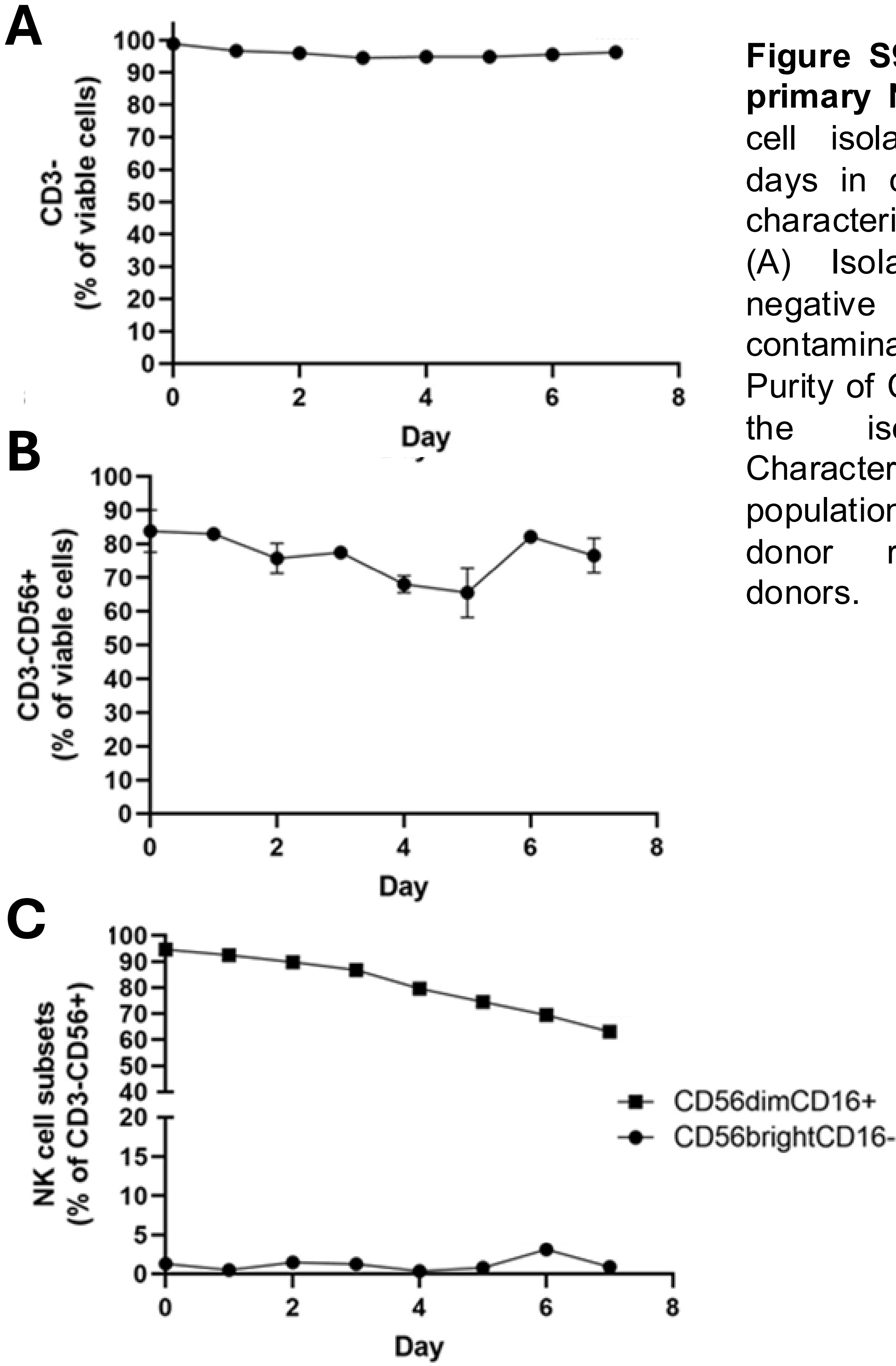

Figure S9. Characterization of primary NK cells. After primary cell isolation and subsequent days in culture, NK cells were characterized via flow cytometry. (A) Isolated NK cells were negative for CD3 and free of contaminating CD3+ T cells. (B) Purity of CD3-CD56+ NK cells or the isolated cells. (C) Characterization of the NK cell populations. Results are one donor representative of 3 donors.

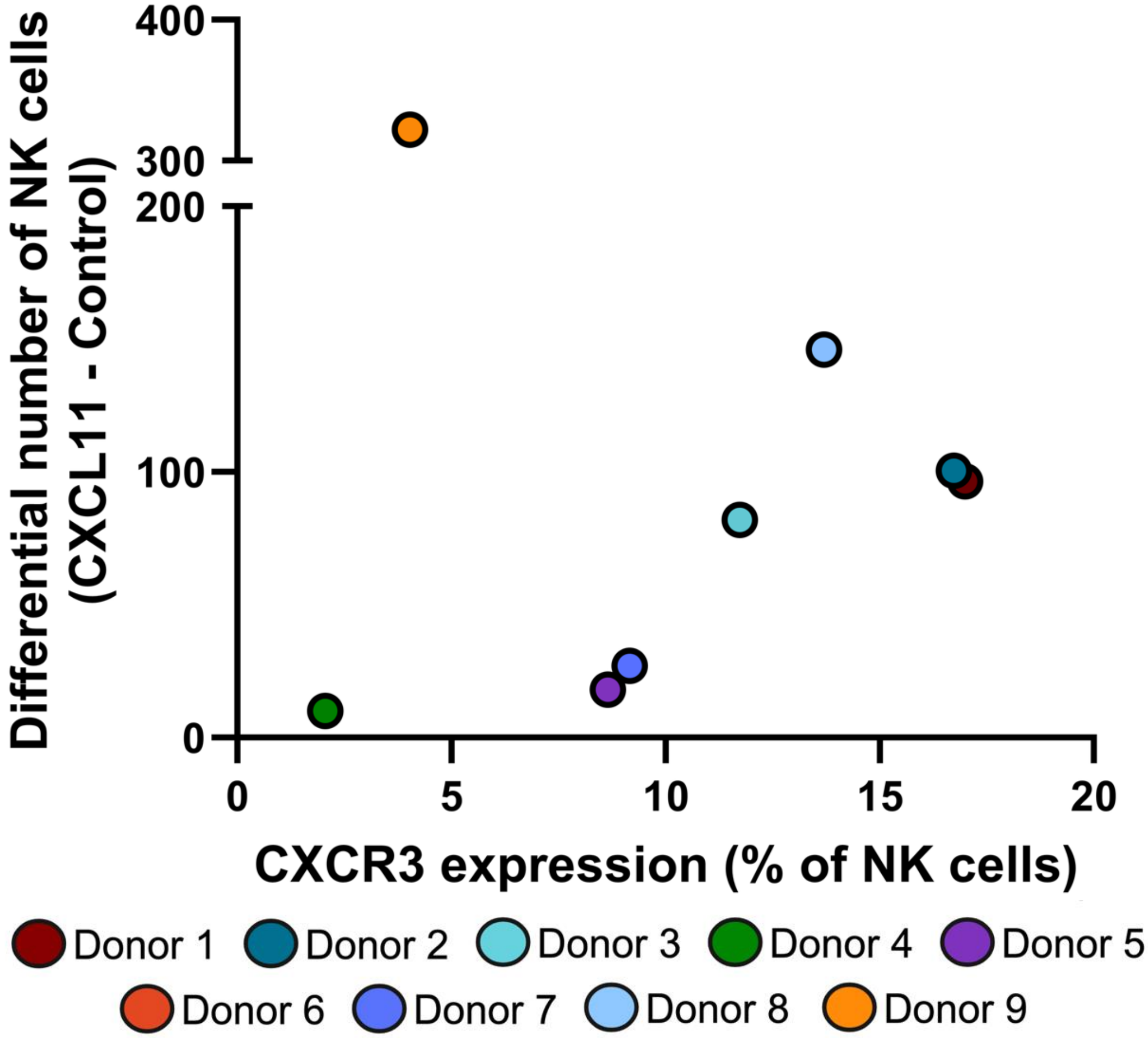

Figure S10. Correlation between the percent of CXCR3 expressing NK cells and the number of NK cells in the gel at day 5 in the CXCL11 chambers minus the NK cells in the medium (control) chambers. Of note, donor 6 was dropped from this analysis as this was the only donor to have an increase in the number of NK cells in the gel at day 5 in the medium (control) chamber compared to the CXCL11 chamber.

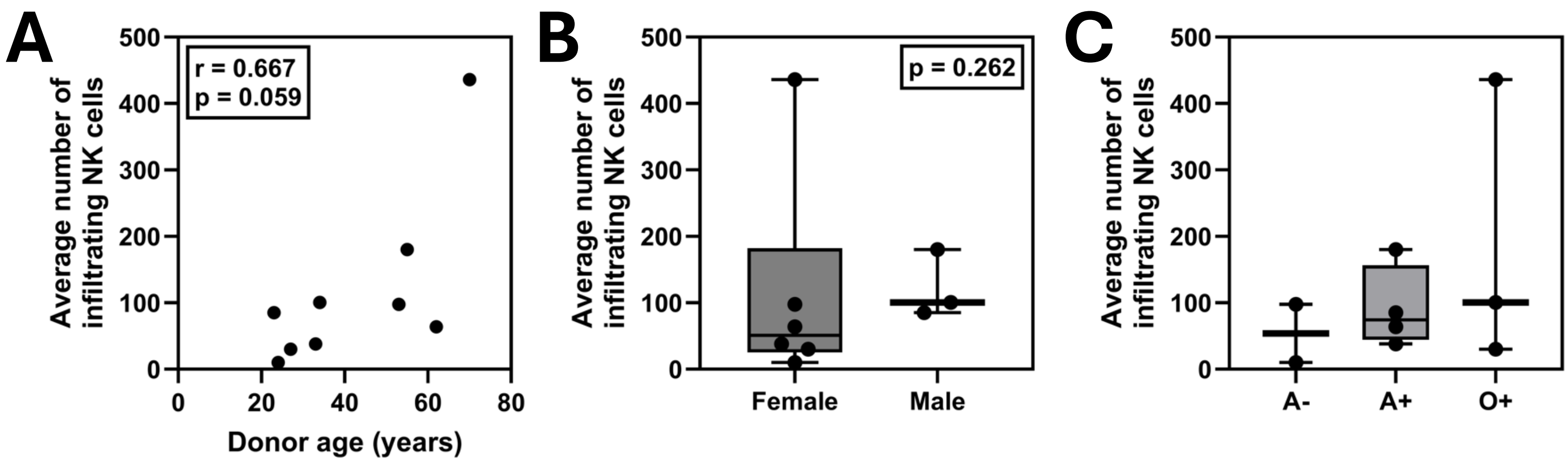

Figure S11. Exploratory analysis of donor characteristics and NK cell migration. NK cell migration efficiency and inhibitor sensitivity were compared with donor demographic variables including (A) age, (B) biologic sex, and (C) ABO blood type. For age, a Spearman correlation was calculated due to insufficient group sizes.

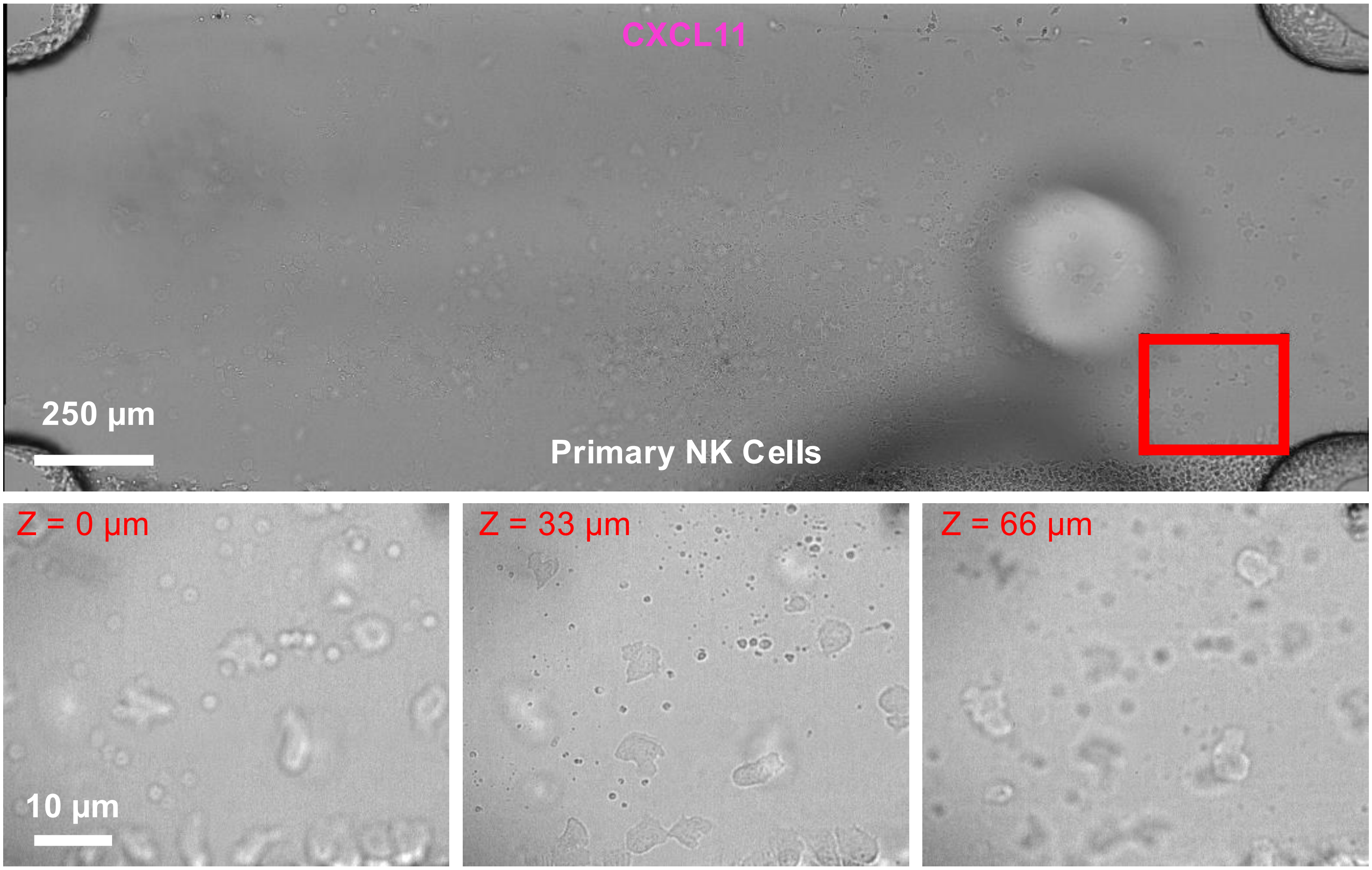

Figure S12. 3D migration of NK cells in the observation chamber of the chemotaxis chip. Representative image of the observation chamber within the chemotaxis chip, which has a total height of 70 µm. The lower panels show magnified views of the red-boxed region, highlighting NK cells located at different z-planes, illustrating their distribution and migration in 3D.
